# miR-214-3p-Sufu-GLI1 is a novel regulatory axis controlling inflammatory smooth muscle cell differentiation from stem cells and neointimal hyperplasia

**DOI:** 10.1186/s13287-020-01989-w

**Published:** 2020-11-03

**Authors:** Shiping He, Feng Yang, Mei Yang, Weiwei An, Eithne Margaret Maguire, Qishan Chen, Rui Xiao, Wei Wu, Li Zhang, Wen Wang, Qingzhong Xiao

**Affiliations:** 1 Centre for Clinical Pharmacology, William Harvey Research Institute, Barts and The London School of Medicine and Dentistry, Queen Mary University of London, London, EC1M 6BQ UK; 2 Institute of Bioengineering, School of Engineering and Materials Science, Queen Mary University of London, London, EC1M 6BQ UK; 3 Department of Cardiology, The First Affiliated Hospital, School of Medicine, Zhejiang University, 79 Qingchun Road, Hangzhou, 310003 Zhejiang China; 4 Department of Cardiology, and Institute for Cardiovascular Development and Regenerative Medicine, Xinhua Hospital Affiliated to Shanghai Jiaotong University School of Medicine, Shanghai, 200092 China; 5 Key Laboratory of Cardiovascular Diseases at The Second Affiliated Hospital, School of Basic Medical Sciences, Guangzhou Medical University, Xinzao Town, Panyu District, Guangzhou, Guangdong, 511436 China; 6 Guangzhou Municipal and Guangdong Provincial Key Laboratory of Protein Modification and Degradation, School of Basic Medical Sciences, Guangzhou Medical University, Xinzao Town, Panyu District, Guangzhou, 511436 Guangdong China

**Keywords:** Adventitia stem/progenitor cells, Neointima formation, Inflammatory smooth muscle cell, Smooth muscle cell differentiation, MicroRNA-214, MicroRNAs, Suppressor of fused (Sufu)

## Abstract

**Background:**

Inflammatory smooth muscle cells (iSMCs) generated from adventitial stem/progenitor cells (AdSPCs) have been recognised as a new player in cardiovascular disease, and microRNA-214-3p (miR-214-3p) has been implicated in mature vascular SMC functions and neointimal hyperplasia. Here, we attempted to elucidate the functional involvements of miR-214-3p in iSMC differentiation from AdSPCs and unravel the therapeutic potential of miR-214-3p signalling in AdSPCs for injury-induced neointimal hyperplasia.

**Methods:**

The role of miR-214-3p in iSMC differentiation from AdSPCs was evaluated by multiple biochemistry assays. The target of miR-214-3p was identified through binding site mutation and reporter activity analysis. A murine model of injury-induced arterial remodelling and stem cell transplantation was conducted to study the therapeutic potential of miR-214-3p. RT-qPCR analysis was performed to examine the gene expression in healthy and diseased human arteries.

**Results:**

miR-214-3p prevented iSMC differentiation/generation from AdSPCs by restoring sonic hedgehog-glioma-associated oncogene 1 (Shh-GLI1) signalling. Suppressor of fused (Sufu) was identified as a functional target of miR-214-3p during iSMC generation from AdSPCs. Mechanistic studies revealed that miR-214-3p over-expression or Sufu inhibition can promote nuclear accumulation of GLI1 protein in AdSPCs, and the consensus sequence (GACCACCCA) for GLI1 binding within smooth muscle alpha-actin (SMαA) and serum response factor (SRF) gene promoters is required for their respective regulation by miR-214-3p and Sufu. Additionally, Sufu upregulates multiple inflammatory gene expression (IFNγ, IL-6, MCP-1 and S100A4) in iSMCs. In vivo, transfection of miR-214-3p into the injured vessels resulted in the decreased expression level of Sufu, reduced iSMC generation and inhibited neointimal hyperplasia. Importantly, perivascular transplantation of AdSPCs increased neointimal hyperplasia, whereas transplantation of AdSPCs over-expressing miR-214-3p prevented this. Finally, decreased expression of miR-214-3p but increased expression of Sufu was observed in diseased human arteries.

**Conclusions:**

We present a previously unexplored role for miR-214-3p in iSMC differentiation and neointima iSMC hyperplasia and provide new insights into the therapeutic effects of miR-214-3p in vascular disease.

**Supplementary information:**

**Supplementary information** accompanies this paper at 10.1186/s13287-020-01989-w.

## Introduction

Smooth muscle cell (SMC) accumulation and phenotypic transition are critical steps for vascular remodelling in response to vascular injury. Although recent ground-breaking efforts, using SMC-restricted lineage-tracking techniques, have provided definitive evidence to support the conventional concept that medium SMCs are the main cellular origins of (neo)intimal SMCs upon injury [1–3], other studies also show that vascular stem/progenitor cells (SPCs), particularly SPCs located in the adventitial layer (namely AdSPCs), represent an additional source of the (neo)intimal SMCs in the atheroma [4, 5]. There is compelling evidence to show that the blood vessel walls contain resident SPCs, such as smooth muscle progenitor cells that can migrate into the intima, where they differentiate into SMCs and contribute to atherosclerotic/neointima lesion formation and plaque stabilisation [6–12]. It is now widely acknowledged that inflammatory SMCs (iSMCs), co-expressing inflammatory cell markers and immature SMC markers and derived from bone marrow progenitor cells and/or vessel residential SPCs, are one of the major cellular sources of neointimal hyperplasia in severe vessel injury models [13–16]. Therefore, investigations into the mechanisms controlling iSMC differentiation from SPCs are essential for us to fully understand the pathogenesis of vascular diseases and remodelling and thus are of great clinical importance.

MicroRNAs (miRNAs) are endogenous, highly conserved single short strands of non-coding RNA. They have been reported as important regulators for stem cell differentiation, cardiovascular development and cardiac regeneration and are emerging as novel therapeutics as well as clinical biomarkers for cardiovascular diseases, including restenosis after vascular injury or stent implantation [17] and atherosclerosis. Although a handful miRNAs including miR-22 [18, 19], miR-34a [20, 21], miR-124 [22] and miR-143/145 cluster [23, 24] have recently been reported to play a role in modulating SMC differentiation from SPCs and controlling multiple processes of vascular diseases, further extensive investigations are warranted to fully elucidate the potential involvement of each miRNA in SMC differentiation and their significance in healthy and diseased arteries.

In particular, miR-214-3p has been reported to be highly expressed at early murine embryonic stages (E10.5~E12.5) in the heart [25], vasculature-rich adult organs and tissues [25, 26], differentiating SMCs [20], vascular endothelial cells and SMCs [26]. Functionally, miR-214-3p has been implicated in skeletal muscle differentiation [27–29], angiogenesis [26, 30], heart protection from ischemic injury [25], bone formation [31], arterial remodelling [32] and pathological perivascular fibrosis in hypertension [33]. The aforementioned studies describe a developmental and pathological role for miR-214-3p in the cardiovascular system, but as of yet, no report studying its regulatory role in the iSMC generation from AdSPCs within the context of injury-induced arterial remodelling has been carried out. Here, we report a novel role for miR-214-3p in governing iSMC generation from AdSPCs in the context of neointimal SMC hyperplasia.

## Materials and methods

### Murine aortic AdSPC isolation, SMC or iSMC differentiation from AdSPCs

The procedures for mouse aortic AdSPC isolation and culture are described in our previous study [6]. Undifferentiated AdSPCs (p3~p10) were cultured in SMC differentiation induction medium (DMEM supplemented with 5% FBS and 5 ng/ml TGFβ1) in the absence (for SMC differentiation) or presence (for iSMC differentiation) of 50 ng/ml TNFα for 2 to 6 days. The medium was refreshed every other day.

### Mouse femoral artery denudation injury and perivascular delivery of miR-214-3p agomiRs and neointima cell isolation

The femoral artery denudation injury murine model has been widely used in preclinical studies to replicate the pathological processes of human percutaneous transluminal angioplasty [34, 35]. Anesthetised C57BL/6 mice (8-week-old, male, 20~22 g, Charles River Laboratories) each underwent the wire-induced denudation injury surgical procedure as described previously [18, 21, 32]. For perivascular delivery of miR-214-3p, wire-injured femoral arteries were randomly assigned to a miR-214 or Cel-miR-67 agomiRs group for application with pluronic gel as described in our previous studies [18, 21, 32]. Briefly, after wire injury, 100 μl of 30% pluronic gel containing chemically modified and cholesterol-conjugated 2.5 nmol miR-214-3p or scrambled (Cel-miR-67) agomiRs was applied perivascularly to injured femoral arteries. The micrON™ miRNA agomiRs were purchased from RiboBio (Guangzhou RiboBio Co., Ltd., China).

Procedures for cell isolation from the injured femoral arteries were similar to the protocols described in the previous studies [36–38]. Two weeks after injury and miRNA treatment, injured femoral arteries (5~10 mm) were dissected out for cell isolation using a similar protocol to AdSPC isolation as described previously [36–38]. The isolated single cells were re-suspended in SMC medium (DMEM with 10% FBS, and 1% P/S) and transferred to six-well culture plate (for RNA isolation) or chamber slides (for immunostaining) pre-coated with 0.04% gelatin solution for up to 4 days prior to analysis.

### Mouse femoral artery denudation injury and perivascular transplantation of AdSPCs

Eight-week-old male C57BL/6 mice (20~22 g) purchased from Charles River Laboratories were subjected to arterial injury and AdSPC transplantation. Briefly, arterial injury was induced by an endothelial denudation procedure as previously described [18, 21, 32]. Immediately after injury, 100 μl Matrigel mixed with 20 μl of cell culture medium (Vehicle control), or 1 × 10^6^ AdSPCs transfected with control AgomiR (AdSPCs/Cel-miR-67) or miR-214-3p AgomiR (AdSPCs/miR-214), was randomly applied perivascularly to the injured femoral arteries. The injured arteries (~ 5.0 mm) were harvested at days 7 and 28 post-injury for gene expression, immunofluorescence staining and morphometric analysis.

Additional materials and methods used in this study are described in detail in the Online Supporting Information, including the protocols for AdSPC differentiation; morphometric analysis of arterial remodelling; lentiviral construction and infection; plasmid construction, transient transfection and luciferase assay; miRNA transfection; ELISA analysis of S100A4 and other cytokines; real-time quantitative PCR (RT-qPCR); immunoblotting; indirect immunofluorescent staining for cells; and chromatin immunoprecipitation (ChIP) assays.

### Statistical analysis

Animals were randomly allocated to their experimental groups. Data collection and evaluation of all experiments were performed blinded to the group identity. The results are presented as mean ± standard error of the mean (SEM). Statistical analysis was performed using GraphPad Prism (v8.3, GraphPad Software, USA). The Shapiro-Wilk normality test was used for checking the normality of the data. Two-tailed unpaired Student’s *t* test was used for comparisons between 2 groups, or one-way analysis of variance with a post hoc test of Tukey’s analysis was applied when more than two groups were compared if the data displayed a normal distribution. Conversely, non-parametric Mann-Whitney *U* test was used for comparing two groups, if the data did not display normal distribution or if the number of observations from each group was smaller than 5 (*n* < 5). Spearman’s rank correlation analyses were conducted to characterise the relationships between the gene expression levels of miR-214-3p and Sufu in human arterial specimens. Alpha = 0.05 was chosen as the significance level, and a value of *P* < 0.05 was considered as statistically significant.

### Data availability

Some data may not be made available because of privacy or ethical restrictions. All remaining data are contained within the article. Other detailed materials and methods were provided in the Online Supporting Information.

## Results

### A role for miR-214-3p in SMC differentiation from AdSPCs

Isolated AdSPCs grew into colonies with a uniform morphology (Figure S1A) and were positive for the typical AdSPC markers Sox10, Sox17 and Nestin, but negative for other cell lineage markers (Figure S1B). Flow cytometry analysis showed that over 92% of these cells were double positive for Sox10 and Nestin (Figure S1C). RT-qPCR data showed that the respective expression levels of AdSPC marker and SMC genes were significantly decreased and increased in AdSPCs at a later stage (P12) (Figure S1D), suggesting that the AdSPCs can be maintained in vitro for a long period (up to 10 passages, P10) without apparent change of the gene expression. Therefore, AdSPCs between P3 and P10 were used in this study. As expected, AdSPCs were successfully induced to differentiate towards SMCs in response to TGFβ1 (Figure S2). To study the potential involvement of miRNAs in SMC differentiation from AdSPCs, we first detected if the expression levels of miR-22, miR-34a and miR-214, the three top modulated miRNAs during SMC differentiation from embryonic stem cells [20], were altered in this process and found that miR-214-3p is the most upregulated miRNA among them in this SMC differentiation model (Figure S3A). miR-214-3p gain/loss-of-function experiments were conducted in AdSPCs to confirm a role for miR-214-3p in SMC differentiation from AdSPCs. As expected, miR-214-3p expression in AdSPCs was successfully upregulated and downregulated by miR-214-3p mimics and inhibitor, respectively (Figure S3B). Importantly, gene expression profiles showed that all four SMC genes examined here were significantly upregulated by miR-214-3p over-expression but downregulated by miR-214-3p inhibition (Figure S3C). These findings were confirmed at the protein level by Western blot assays (Figure S3D & S3E), supporting a role for miR-214-3p in regulating SMC differentiation from AdSPCs.

### miR-214-3p inhibits TNFα-induced inflammatory SMC differentiation from AdSPCs

**Fig. 1:**
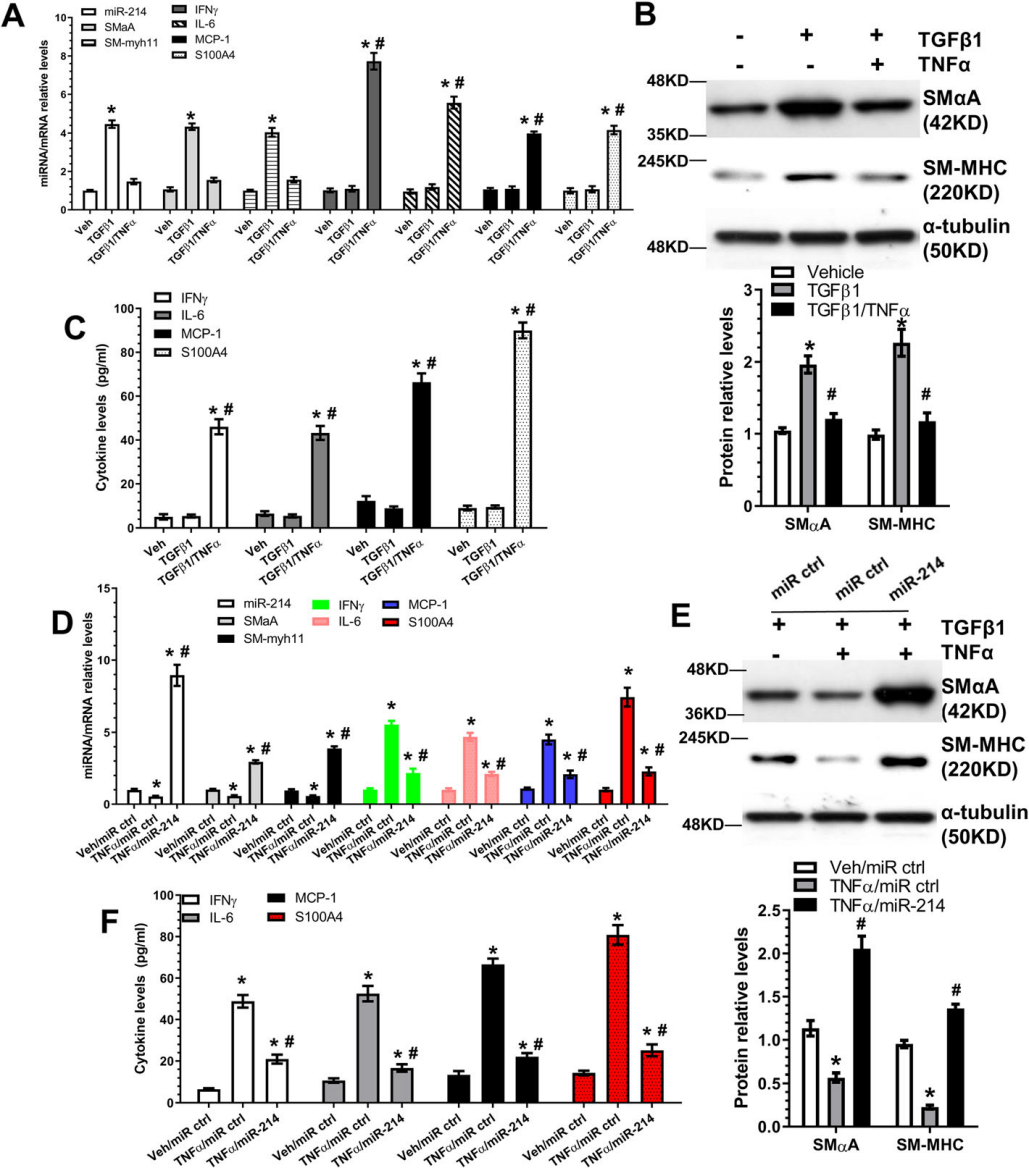
miR-214-3p inhibits TNFα-induced inflammatory SMC differentiation from AdSPCs. **a**–**c** Passage 3–8 AdSPCs were cultured in SMC differentiation medium in the absence (vehicle (Veh)) or presence of 50 ng/ml TNFα for 4 days to allow for inflammatory SMC (iSMC) differentiation. Total RNA, protein and conditioned culture medium were harvested and subjected to RT-qPCR (**a**), Western blot (**b**) and ELISA analyses (**c**), respectively. **d**–**f** miR-214-3p over-expression rescued mature/contractile SMC marker expressions and inhibited TNFα-induced inflammatory cytokine gene expression. AdSPCs were differentiated into iSMCs (DMEM containing 5 ng/ml TGFβ1 and 50 ng/ml TNFα) for 4 days, then transfected with miR-214-3p mimics (miR-214) or negative control (miR ctrl) and cultured for 48 h in the same culture medium. Total RNA, proteins and conditioned culture medium were harvested and subjected to RT-qPCR (**d**), Western blot (**e**) and ELISA (**f**) analysis, respectively. The data presented here are representative (top panels in **b** and **e**) or mean ± S.E.M. (**a**, **c**, **d**, **f** and bottom panels in **b** and **e**) of five or six independent experiments (*n* = 5 or 6). *P < 0.05 (vs vehicle/control); ^#^*P* < 0.05 (TGFβ1/TNFα vs TGFβ1 or TNFα/miR-214 vs TNFα/miR ctrl) (one-way ANOVA with a post hoc test of Tukey’s analysis). miR-214 indicates miR-214-3p

Vascular SMCs can switch to a ‘pro-inflammatory’ phenotype, or inflammatory SMC (iSMC), in response to vascular injury, which promotes monocyte/macrophage recruitment into the vascular wall and triggers the pathological processes of atherosclerosis and arterial remodelling [39]. To mimic iSMC differentiation/generation from AdSPCs in vitro, differentiating AdSPCs were incubated with the pro-inflammatory cytokine, TNFα. It is worth mentioning that TNFα alone failed to induce iSMC differentiation from AdSPCs in vitro; therefore, TNFα and TGFβ1 were used together to generate iSMCs in the following experiments. SMC differentiation was successfully induced in the presence of TGFβ1, as evidenced by the induction of two SMC-specific genes (SMαA and SM-myh11), while no significant induction was observed with inflammatory genes (IFNγ, IL-6 and MCP-1/CCL2) and S100A4, a reported marker for the rhomboid SMC (R-SMC) phenotype [40]. Interestingly, with the addition of TNFα, the differentiating AdSPCs were primed to adopt an iSMC phenotype, whereby decreased expression levels of SMC genes and increased expression levels of the inflammatory genes and S100A4 were observed (Fig. 1a–c).

Multiple functional assays were conducted in mature SMCs with different phenotypes (serum-starved, ‘synthetic’ and ‘contractile’ SMCs) and iSMCs to further characterise the iSMC phenotype. Compared to ‘synthetic’ SMCs, iSMCs displayed a trend of increased SMC gene expression with a significant drop in the expression of extracellular matrix genes (Figure S4A). Among the cells, iSMCs expressed the highest level of inflammatory genes and produced the largest amount of inflammatory cytokines (Figure S4A & S4B). Similarly, we observed the highest level of NFκB activation in iSMCs (Figure S4C) compared to the other groups. Functionally, iSMCs grew and migrated faster than ‘contractile’ SMCs but slower than ‘synthetic’ SMCs (Figure S4D & S4E). These data have collectively demonstrated that iSMCs display a distinct phenotype with moderate levels of matrix gene expression, cell proliferation and migration but a high level of inflammation, compared to either ‘synthetic’ or ‘contractile’ SMCs.

Importantly, we observed an increased expression level of miR-214-3p induced by TGFβ1 alone, and this was inhibited once TNFα was added into the differentiating AdSPCs (Fig. 1a), suggesting a role for miR-214-3p in these processes. Indeed, RT-qPCR data showed that while TNFα incubation significantly inhibited miR-214-3p expression, transfection with miR-214-3p mimics significantly increased its expression (Fig. 1d). Crucially, we observed a similar trend with SMC gene expression to miR-214-3p expression, but an opposite trend for inflammatory genes and S100A4 (Fig. 1d-f), supporting a notion that miR-214-3p can rescue the iSMC phenotype induced by inflammation.

### Sonic hedgehog-glioma-associated oncogene 1 signal activation and modulation by miR-214-3p during iSMC differentiation

**Fig. 2:**
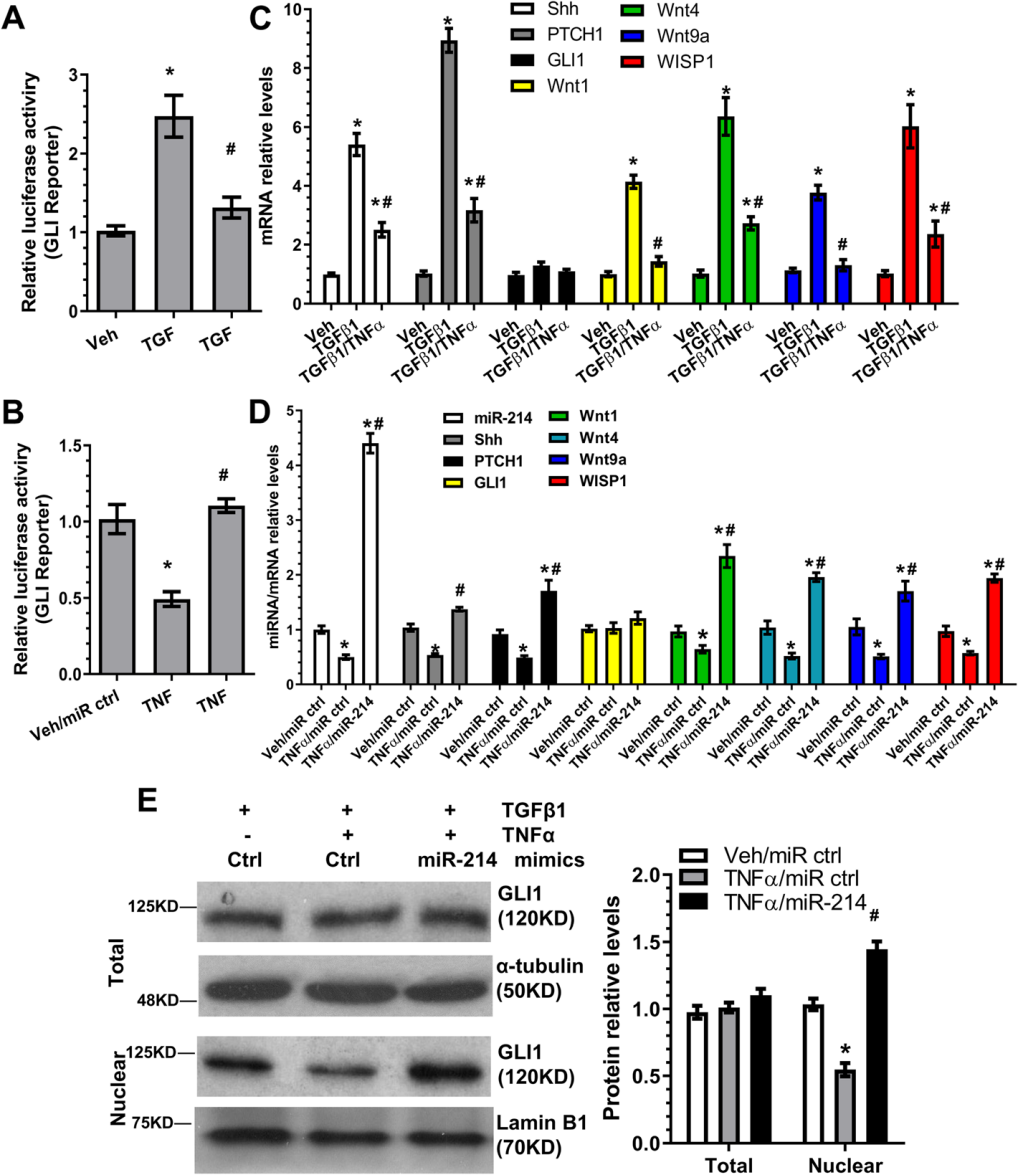
Sonic hedgehog-glioma-associated oncogene 1 (Shh-GLI1) signal activation and modulation by miR-214-3p during iSMC differentiation. **a**, **b** Luciferase activity assays to examine Shh-GLI signalling activity. AdSPCs were transfected with GLI reporter constructs and subjected to various treatments indicated in respective figures. Two days later, total cell lysates were harvested and subjected to luciferase activity assays. **c**, **d** RT-qPCR detection of Shh-GLI target gene expression. **e** miR-214-3p promotes GLI1 nuclear translocation. AdSPCs were subjected to similar treatments described in Fig. 1**d**–**f**. Total cell lysate and nuclear proteins were extracted and subjected to Western blot analysis. The data presented here are representative (left panel in **e**) or mean ± S.E.M. (**a**–**d** and right panel in **e**) of five independent experiments (*n* = 5). *P < 0.05 (vs vehicle/control); ^#^*P* < 0.05 (TGFβ1/TNFα vs TGFβ1 or TNFα/miR-214 vs TNFα/miR ctrl) (one-way ANOVA with a post hoc test of Tukey’s analysis). miR-214 indicates miR-214-3p

Sonic hedgehog-glioma-associated oncogene 1 (Shh-GLI1) signalling has been extensively implicated in adventitial smooth muscle progenitor cell self-renewal and differentiation [41, 42] and blood vessel formation [43]. GLI1 reporter activity assays revealed that Shh-GLI1 signalling was significantly activated in TGFβ1-induced SMC differentiation, but its activity was almost completely abolished in iSMCs (Fig. 2a). Additionally, over-expression of miR-214-3p in iSMCs could reverse GLI1 reporter activity (Fig. 2b), suggesting that miR-214-3p can modulate the Shh-GLI1 signalling pathway during iSMC differentiation from AdSPCs. This was further supported by gene expression profiles, in which our data showed that all three main components of the Shh-GLI1 signalling pathway (Shh, the transmembrane receptor [PTCH1] and the transcriptional effector [GLI1]), as well as its downstream target genes (Wnt1, Wnt4, Wnt9a and WISP1) were significantly activated by TGFβ1 treatment. These effects were blunted by the inclusion of TNFα into the differentiating AdSPCs (Fig. 2c). Moreover, the reverse phenomenon was observed once miR-214-3p mimics were introduced into iSMCs (Fig. 2d). Once activated by Shh binding to PTCHs, the smoothened receptor causes GLI1 relocalisation from the cytoplasm to the nucleus, where GLI1 regulates the gene expression through binding to the consensus GLI1-binding element (GACCACCCA) within its target gene promoters [44]. Western blot assay showed that nuclear, but not total, GLI1 protein levels were significantly modulated and regulated by miR-214-3p in iSMCs (Fig. 2e). This was further confirmed by immunofluorescence staining (Figure S5). Taken together, this data demonstrates that the Shh-GLI1 signalling pathway is closely modulated by miR-214-3p during iSMC differentiation/generation from AdSPCs.

### Identification of Sufu as the target gene of miR-214-3p during iSMC differentiation from AdSPCs

**Fig. 3:**
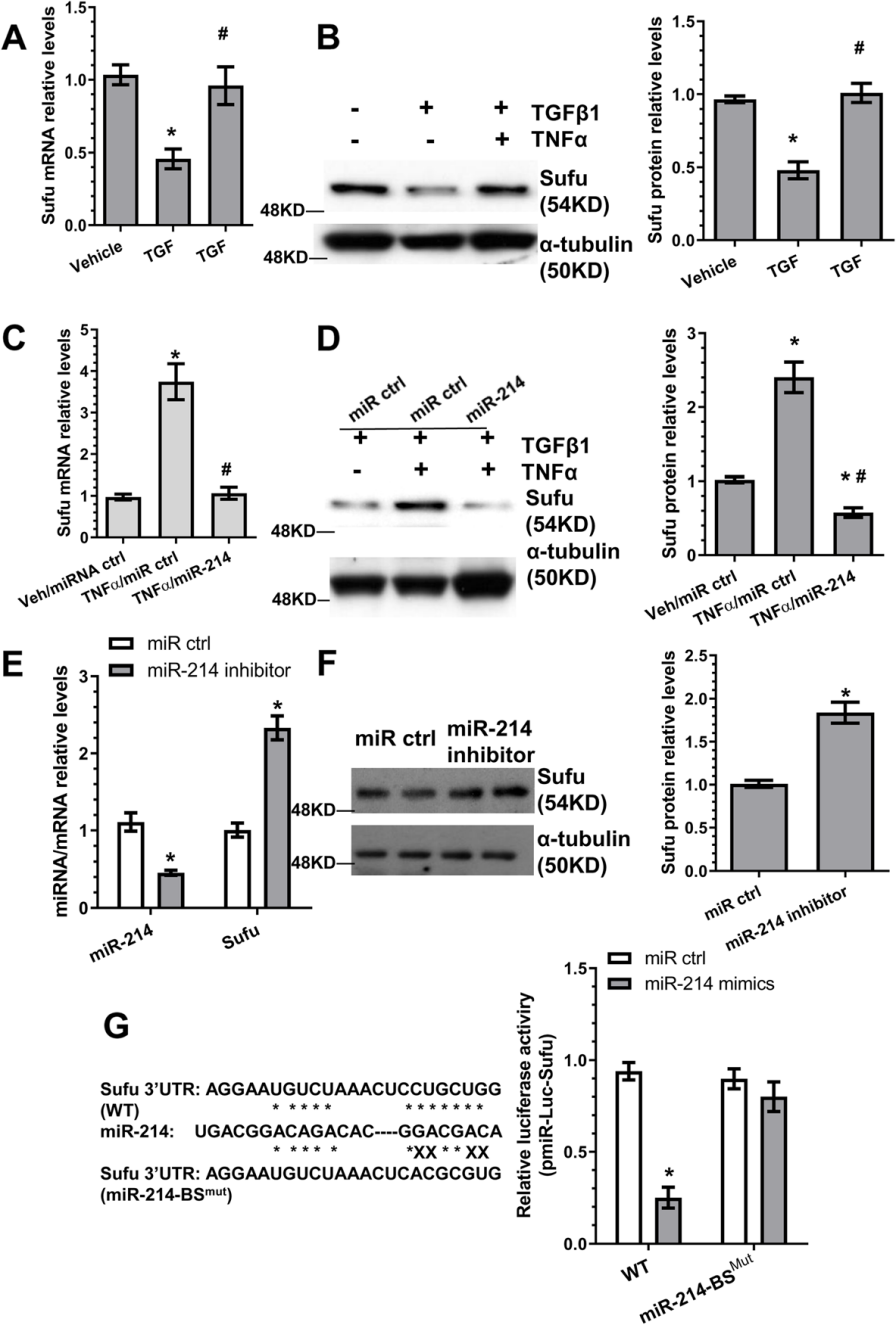
Identification of Sufu as the target gene of miR-214-3p during TNFα-induced iSMC differentiation from AdSPCs. **a**, **b** Sufu is closely modulated during iSMC differentiation from AdSPCs. **c**, **d** miR-214-3p over-expression in iSMCs inhibits Sufu gene expression. **e**, **f** Upregulation of Sufu by miR-214-3p inhibition. AdSPCs were differentiated into iSMCs for 4 days, then transfected with miR-214-3p inhibitor or negative control (miR ctrl) and cultured for 48 h in the same culture medium. Total RNAs and proteins were harvested and subjected to RT-qPCR (**e**) and Western blot (**f**) analysis, respectively. **g** The binding site is required for miR-214-3p-mediated Sufu gene repression. The potential wild-type binding site (WT) for miR-214-3p within Sufu gene 3′UTR and its mutant (BS^mut^) are depicted in this illustration (left). miR-214-3p mimics or negative control (miR ctrl) was co-transfected into iSMCs with wild-type Sufu 3′UTR reporter (WT) or miR-214-3p binding site mutants [miR-214BS^mut^], respectively. Luciferase activity assay was measured at 48 h post-transfection. The data presented here are representative (left panels in **b**, **d** and **f**) or mean ± S.E.M. of five (**a**, **c**, **e** and right panels in **b**, **d** and **f**, *n* = 5) or eight (**g**, *n* = 8) independent experiments. *P < 0.05 (vs vehicle/miR ctrl); ^#^*P* < 0.05 (TGFβ1/TNFα vs TGFβ1 or TNFα/miR-214 vs TNFα/miR ctrl). (One-way ANOVA with a post hoc test of Tukey’s analysis for **a**–**d**; unpaired *t* test for **e**–**g**). miR-214 indicates miR-214-3p

Suppressor of fused (Sufu) negatively regulates hedgehog signalling through direct binding of GLI1 and by retaining GLI1 in the cytoplasm [45, 46]. Interestingly, we observed an opposite trend for Sufu (Fig. 3a, b) and miR-214-3p (Fig. 1a) expression during iSMC differentiation from AdSPCs, and that Sufu was negatively regulated by miR-214-3p (Fig. 3c–f) as demonstrated in miR-214-3p over-expression (Fig. 3c, d) and inhibition (Fig. 3e, f) experiments, respectively. Moreover, Sufu has been reported as a target of miR-214-3p in zebrafish [27], and both mouse and human Sufu 3′UTR harbour miR-214-3p binding sites, indicating that Sufu is the mRNA target of miR-214-3p during iSMC differentiation from AdSPCs. This was supported by luciferase data with Sufu 3′UTR luciferase reporter, in which we found that the reporter activity was dramatically supressed by miR-214-3p over-expression, and the conserved miR-214-3p binding site within Sufu 3′UTR is required for Sufu gene suppression by miR-214-3p since the inhibitory effect of miR-214-3p over-expression on Sufu 3′UTR reporter activity was almost completely abolished once the binding site was mutated (Fig. 3g). Taken together, this data clearly demonstrates that Sufu is a true mRNA target of miR-214-3p in the context of iSMC differentiation/generation from AdSPCs.

### Sufu mediates SMC gene expression by modulating GLI1 nuclear translocation

**Fig. 4:**
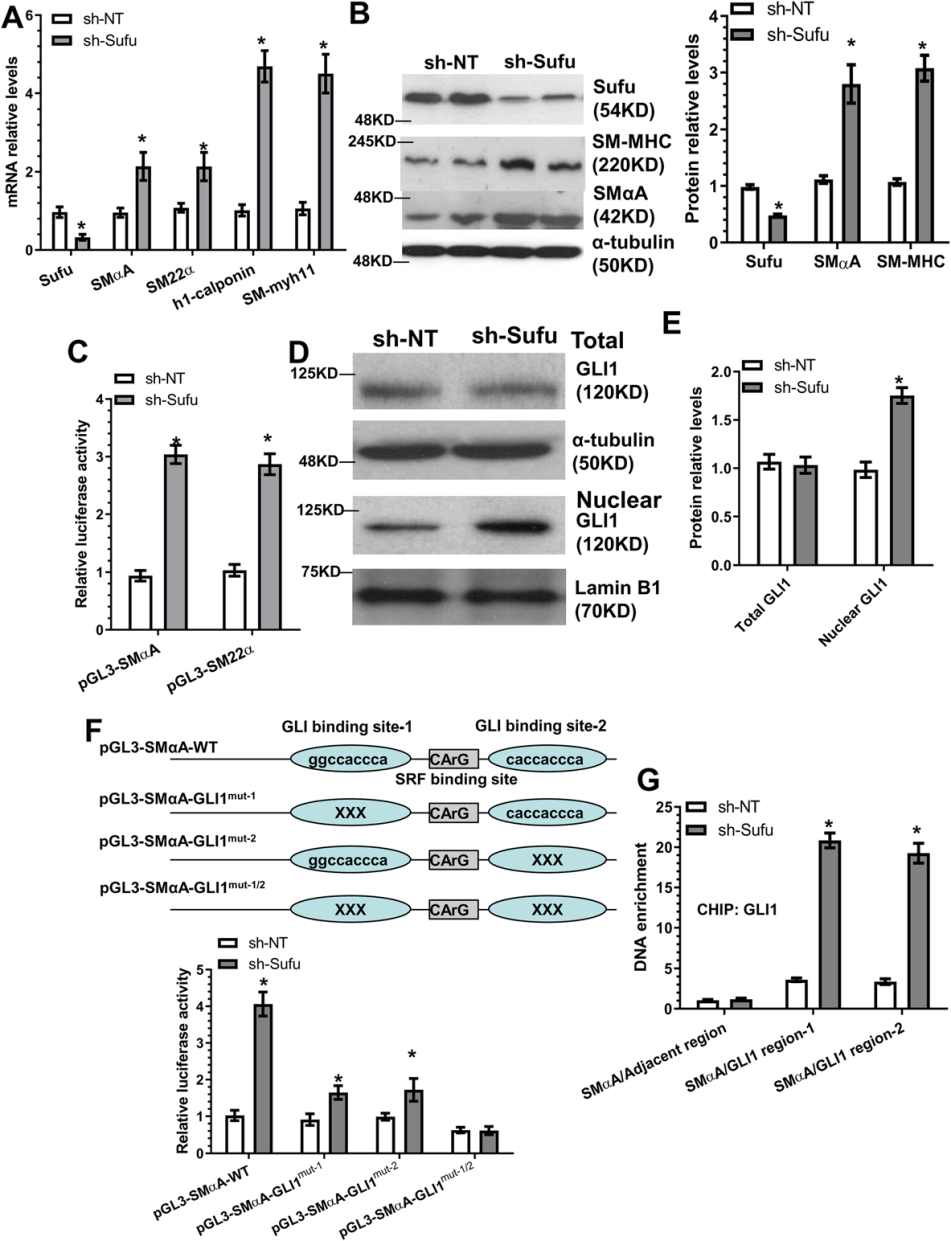
Sufu mediates SMC gene expression by modulating GLI1 nuclear translocation. **a**, **b** Sufu knockdown upregulated SMC gene expression. AdSPCs were infected with non-target (sh-NT) or Sufu (sh-Sufu) shRNA lentivirus, followed by iSMC differentiation. Total RNAs and proteins were harvested and subjected to RT-PCR (**a**) and Western blot (**b**) analyses, respectively. **c** SMC gene promoter activity was upregulated by Sufu inhibition. AdSPCs infected with sh-NT or sh-Sufu lentivirus were transfected with SMC gene promoters (pGL3-SMαA or SM22α). Two days later, total cell lysates were harvested and subjected to luciferase activity assays. **d**, **e** Sufu knockdown increases GLI1 nuclear translocation. AdSPCs were subjected to similar treatments described in **a** and **b**. Total cell lysate and nuclear proteins were extracted and subjected to Western blot analysis. **f** Two GLI1 binding sites within the SMαA gene promoter region are required for SMαA gene upregulation by Sufu knockdown in AdSPCs. The potential wild-type binding site (WT) of GLI1 within the SMαA gene promoter region and its mutants (GLI1^mut^) is depicted in this illustration (top). AdSPCs infected with sh-NT or sh-Sufu lentivirus were transfected with wild-type and mutated SMαA gene promoter reporters as indicated. Luciferase activity assay was measured at 48 h post-transfection. **g** Sufu inhibition increases GLI1 enrichment within the SMαA gene promoter. ChIP assays were performed using antibodies against GLI1 or normal IgG. PCR amplifications of the adjacent regions were included as an additional control for specific promoter DNA enrichment. The data presented here are representative (left panel in **b**, and **d**) or mean ± S.E.M. (**a**, **c**, **e**–**g** and right panel in **b**) of five independent experiments (*n* = 5). *P < 0.05, **P < 0.01 (vs sh-NT, unpaired *t* test)

Compared to the respective control cells, Sufu knockdown significantly upregulated SMC gene expression at both the mRNA (Fig. 4a) and protein (Fig. 4b) levels, while the opposite effect was observed when over-expressing Sufu in the differentiating AdSPCs (Figure S6A), suggesting that Sufu functions as a negative regulator of SMC marker gene expression. This was further confirmed by SMC gene promoter activity assays (Fig. 4c and S6B), indicating that Sufu regulates SMC gene expression at a transcriptional level. RT-qPCR data showed no significant difference for GLI1 mRNA expression levels between control and Sufu knockdown AdSPCs (Figure S6C), while data from both Western blot (Fig. 4d, e) and immunofluorescence staining (Figure S6D) assays revealed that Sufu inhibition in the differentiating AdSPCs results in nuclear accumulation of the GLI1 protein. Interestingly, two GLI1 binding sites were identified within the SMαA gene promoter, and we found that both GLI1 binding sites were important for Sufu-mediated SMαA gene suppression during iSMC differentiation from AdSPCs (Fig. 4f). Finally, chromatin immunoprecipitation (ChIP) assay data revealed that GLI1 binds directly to regions spanning around GLI1-binding element of SMαA gene promoters, and this binding was dramatically enhanced by Sufu inhibition (Fig. 4g). Altogether, the above data suggests that Sufu represses SMC gene expression by modulating the Shh-GLI1 signalling pathway.

### Sufu controls SMC gene expression by regulating the transcription factor, SRF

Luciferase activity assays showed that Sufu knockdown in the differentiating AdSPCs significantly activates SMC gene promoter activities (Figure S7A & S7B) and that SRF binding element (CArG) mutation in SMC gene promoter reporters could almost abrogate their transcriptional activity in response to Sufu inhibition (Figure S7A & S7B), inferring an important role for SRF in Sufu-mediated SMC gene regulation. We found that Sufu over-expression inhibited SRF gene expression (Figure S7C), while Sufu knockdown promoted its expression (Figure S7D). A similar effect was observed in luciferase activity assays with an SRF gene promoter construct (Figure S7E). Importantly, luciferase activity assays, using the SRF gene promoter construct containing the mutated GLI1 binding site, revealed a critical role for the GLI1-binding site within the SRF gene promoter in SRF gene suppression by Sufu (Figure S7F). Moreover, ChIP assays showed a significant enrichment of GLI1 within the SRF gene promoter in AdSPCs with Sufu knockdown (Figure S7G). Taken together, the above findings demonstrate that Sufu regulates SMC gene expression during iSMC differentiation from AdSPCs by modulating the binding of GLI1 to SRF gene promoter and controlling SRF transcriptional activity.

### A regulatory role for the Sufu-GLI1 signal axis in inflammatory gene expression during iSMC differentiation from AdSPCs

**Fig. 5:**
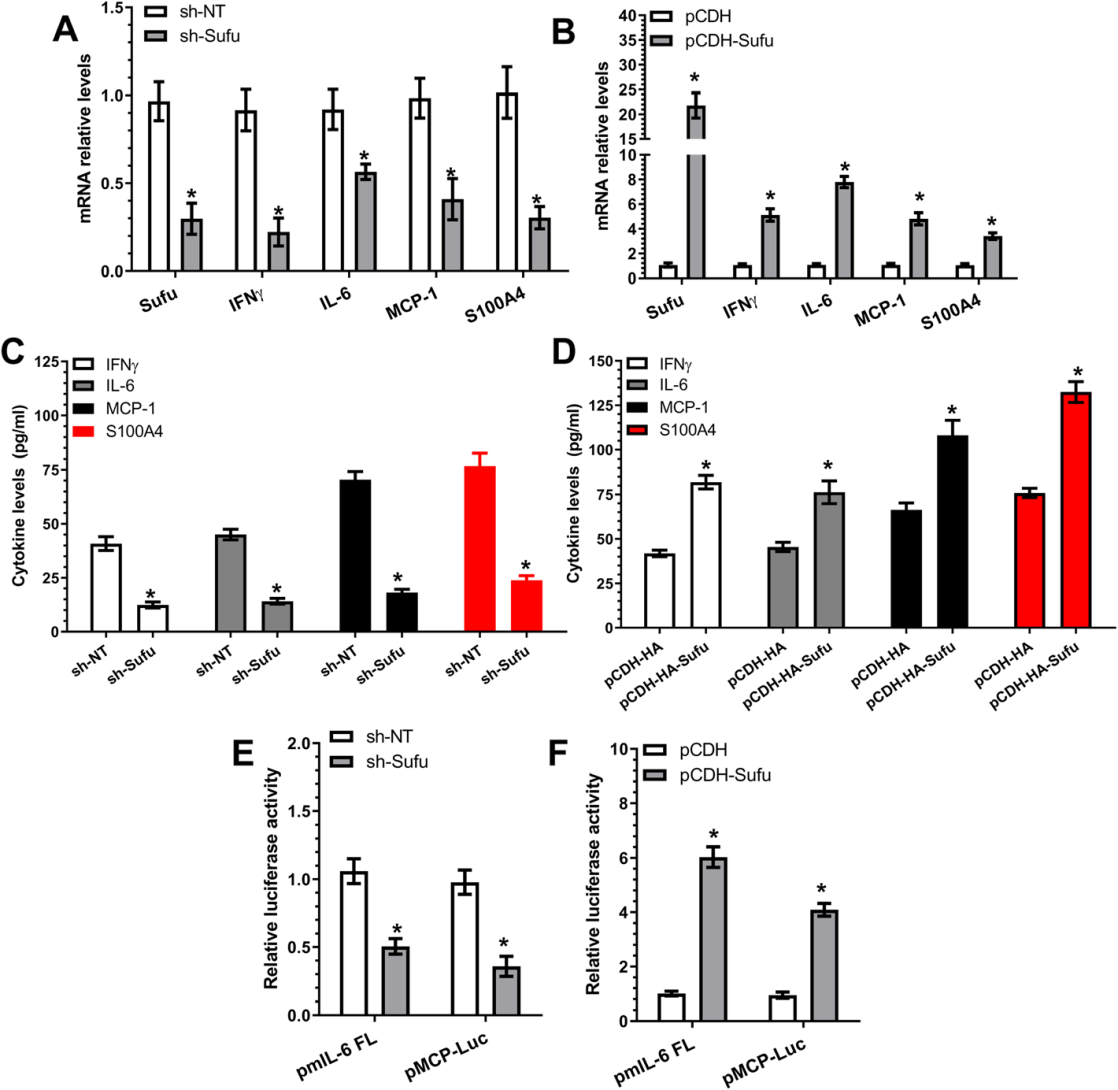
Sufu positively regulates TNFα-induced inflammatory gene expression in AdSPC-derived iSMCs. **a**–**d** Inflammatory gene expression in iSMCs was positively modulated by Sufu. AdSPCs were infected with non-target (sh-NT) or Sufu (sh-Sufu) shRNA lentivirus (**a**, **c**), or control (pCDH) or Sufu over-expressing (pCDH-Sufu) vector (**b**, **d**), followed by iSMC differentiation for 4 days. Total RNAs and conditioned culture medium were harvested and subjected to RT-PCR (**a**, **b**) and ELISA (**c**, **d**) analyses, respectively. **e**, **f** Luciferase activity assays. AdSPCs infected with sh-NT or sh-Sufu (**e**), or pCDH or pCDH-Sufu (**f**) were transfected with IL-6 (pmIL-6 FL) or MCP-1/CCL2 (pMCP-Luc) gene promoter reporters as indicated in the figures, followed by iSMC differentiation. Two days later, total cell lysates were harvested and subjected to luciferase activity assays. The data presented here are mean ± S.E.M. of five (*n* = 5 in **a**, **b**, **e** and **f**) or six (*n* = 6 in **c** and **d**) independent experiments. *P < 0.05 (vs sh-NT or pCDH, unpaired *t* test)

The Sufu-GLI1 signal axis has been reported to play a critical role in the intestinal inflammatory response, and decreased GLI1 activity predisposes to a heightened myeloid response to inflammatory stimuli in inflammatory bowel diseases [47]. We wondered if this axis also played a role in inflammatory gene expression and iSMC differentiation from AdSPCs. RT-qPCR data showed that Sufu inhibition (increased GLI1 activity and nuclear accumulation as shown in Fig. 4d, e) significantly inhibited the expression levels of all three inflammatory genes and S100A4 (Fig. 5a), while Sufu over-expression had the opposite impact (Fig. 5b). A similar effect was confirmed at the protein level in ELISA assays (Fig. 5c, d), as well as at the transcriptional level, as shown by luciferase activity assays with IL-6 and MCP-1/CCL2 gene promoter reporters (pmIL-6 FL and pMCP-Luc) (Fig. 5e, f). Collectively, this data demonstrates that Sufu serves as a positive regulator in inflammatory gene regulation by negatively regulating GLI1 activity during iSMC differentiation from AdSPCs.

### miR-214-3p over-expression in injured arteries decreases iSMC formation

Similar to our previous studies [18, 21, 32], a neointimal layer was observed in the injured arteries treated with Cel-miR-67 agomiRs, while locally enforced expression of miR-214-3p resulted in a smaller neointimal layer (Figure S8A). As expected, cells isolated by enzymatic digestion from the normal medial layer (SMCs/Sham) consistently exhibited a spindle-shaped phenotype with the classic ‘hill-and-valley’ growth pattern at confluence, while cells isolated from the media/neointima layer of the injured arteries displayed a phenotype resembling R-SMCs, as previously reported [48]. Interestingly, miR-214-3p over-expression in the injured arteries reverted the injury-induced R-SMC phenotype to a classic one (Figure S8B). Data from immunofluorescence staining revealed that cells with R-SMC phenotype were positive for SMαA and IL-6, and miR-214-3p over-expression significantly reduced iSMC formation (Figure S8C & S8D). Compared with SMCs, expression levels of miR-214-3p and SMC contractile genes (SMαA and SM-myh11) were significantly decreased, while that of Sufu (miR-214-3p target gene), IL-6 and S100A4 (iSMC markers) were dramatically increased in the neointimal cells treated with control Cel-miR-67 agomiRs (Figure S8E). Importantly, perivascular transfection with miR-214-3p agomiR resulted in an increased level of miR-214-3p in the neointimal cells, which consequently reversed the above-described gene expression profiles (Figure S8E). Altogether, the above data demonstrate that miR-214-3p inhibits iSMC formation within the injured arterial walls.

### Transplantation of AdSPCs increases adverse arterial remodelling in response to injury, while miR-214-3p over-expression in AdSPCs reverses this effect

**Fig. 6:**
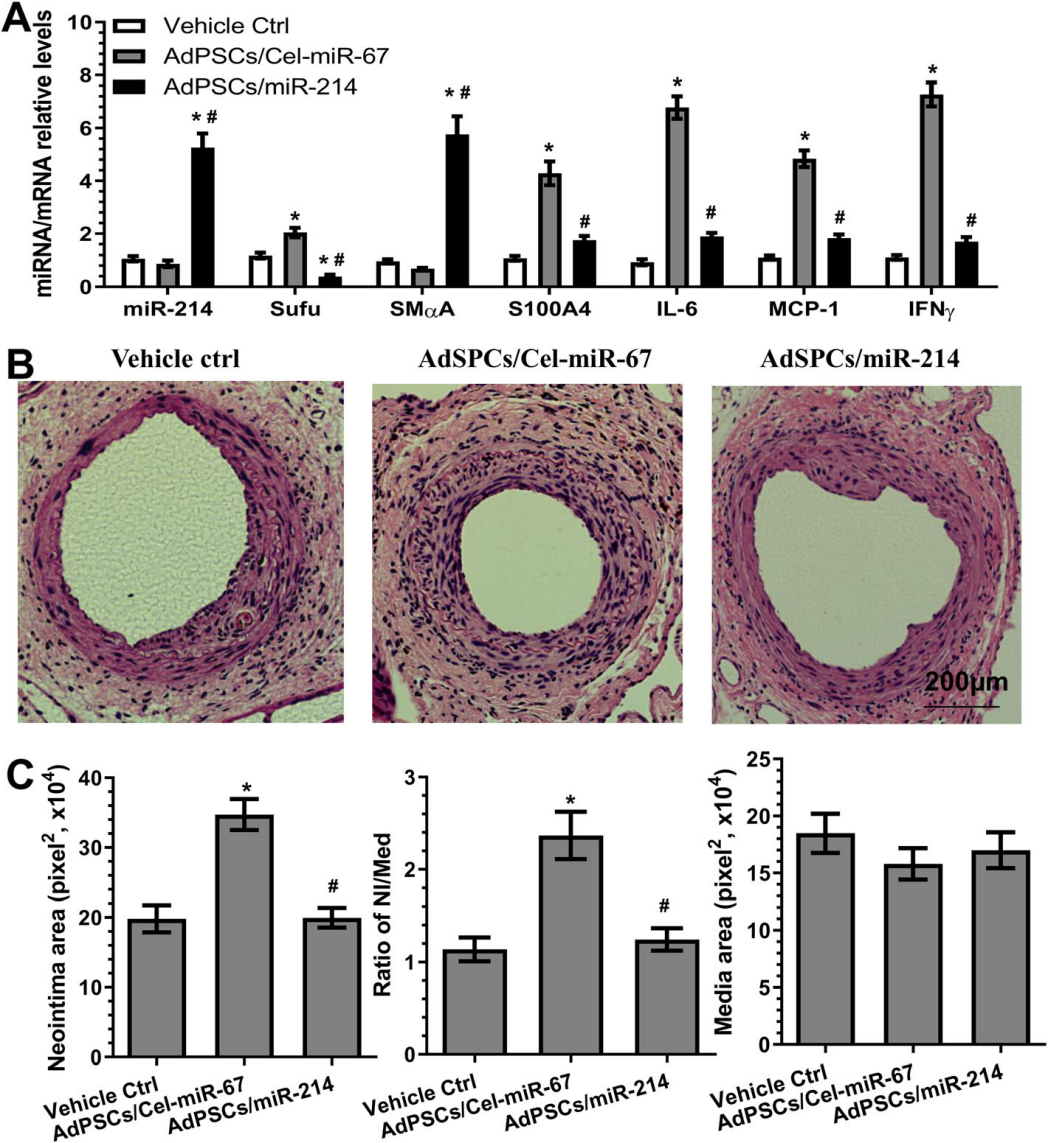
Transplantation of AdSPCs increases adversely arterial remodelling in response to injury, while miR-214-3p over-expression in AdSPCs reverses such processes. Eight-week-old male mice were randomly subjected to arterial injury and AdSPC transplantation. After injury, 100 μl of Geltrex contained culture medium (vehicle ctrl) or 1 × 10^6^ AdSPCs transfected with control AgomiR (AdSPCs/Cel-miR-67) or miR-214-3p AgomiR (AdSPCs/miR-214) was immediately applied and packed around the injured vessel. One (**a**) or 4 (**b**, **c**) weeks later, injured segments of the femoral arteries were harvested and subjected to the following studies as indicated. **a** RT-qPCR detection of gene expressions in the injured arteries. The data presented in **a** are mean ± S.E.M. of five independent experiments (femoral arteries from 3~5 mice were pooled for each experiment, *n* = 5 experiments). **b**, c Morphometric analysis of the wire injury-induced neointima formation. Paraffin sections from the indicated groups (*n* = 10 mice for each group) were prepared and subjected to H&E staining analyses. Representative images (**b**) and morphological characteristics including the neointimal area, neointimal/media (N/M) ratio and media area (**c**) were presented here. *P < 0.05 (vs vehicle Ctrl). ^#^*P* < 0.05 (AdSPCs/miR-214 vs AdSPCs/Cel-miR-67) (one-way ANOVA with a post hoc test of Tukey’s analysis)

Triple immunofluorescence staining of the injured segments of the femoral arteries transplanted with GFP-labelled AdSPCs with antibodies against GFP, SMαA and S100A4 showed that the transplanted AdSPCs could differentiate into iSMCs (cells are positive for GFP, SMαA and S100A4) within the injured arterial walls (Figure S9). To further explore the functional implication of miR-214-3p in controlling iSMC generation from AdSPCs in the context of angioplasty-induced vascular remodelling, 8-week-old male mice were subjected to arterial injury and AdSPC transplantation as indicated in the figures. As previously reported [34, 35], the neointimal lesion was almost undetectable at 1 week following injury; therefore, aortic tissues at 1 week post-injury were harvested and subjected to gene expression analysis. AdSPC transplantation resulted in a trend of decreased miR-214-3p and SMαA expression and significantly increased Sufu, S100A4 and inflammatory gene expression (Fig. 6a). Thus, further supporting previous observations that transplanted AdSPCs can acquire an iSMC phenotype during injury-induced arterial remodelling. Importantly, local transplantation of miR-214-3p over-expressing AdSPCs dramatically increased vascular miR-214-3p and SMαA levels but significantly decreased expression of other genes (Fig. 6a). Consequently, after 28 days, neointimal formation was induced by wire injury of the femoral artery in the mice treated with vehicle control. This was further increased by AdSPC transplantation; however, such advanced arterial remodelling response was prevented in mice transplanted with miR-214-3p over-expressing AdSPCs (Fig. 6b, c). Taken together, our data confirmed a preventive effect of miR-214-3p on neointimal SMC hyperplasia, likely through its inhibitory role in iSMC differentiation/generation from AdSPCs in response to vascular injury.

### Expression profiles of miR-214-3p and Sufu in the healthy and diseased human vessels

**Fig. 7:**
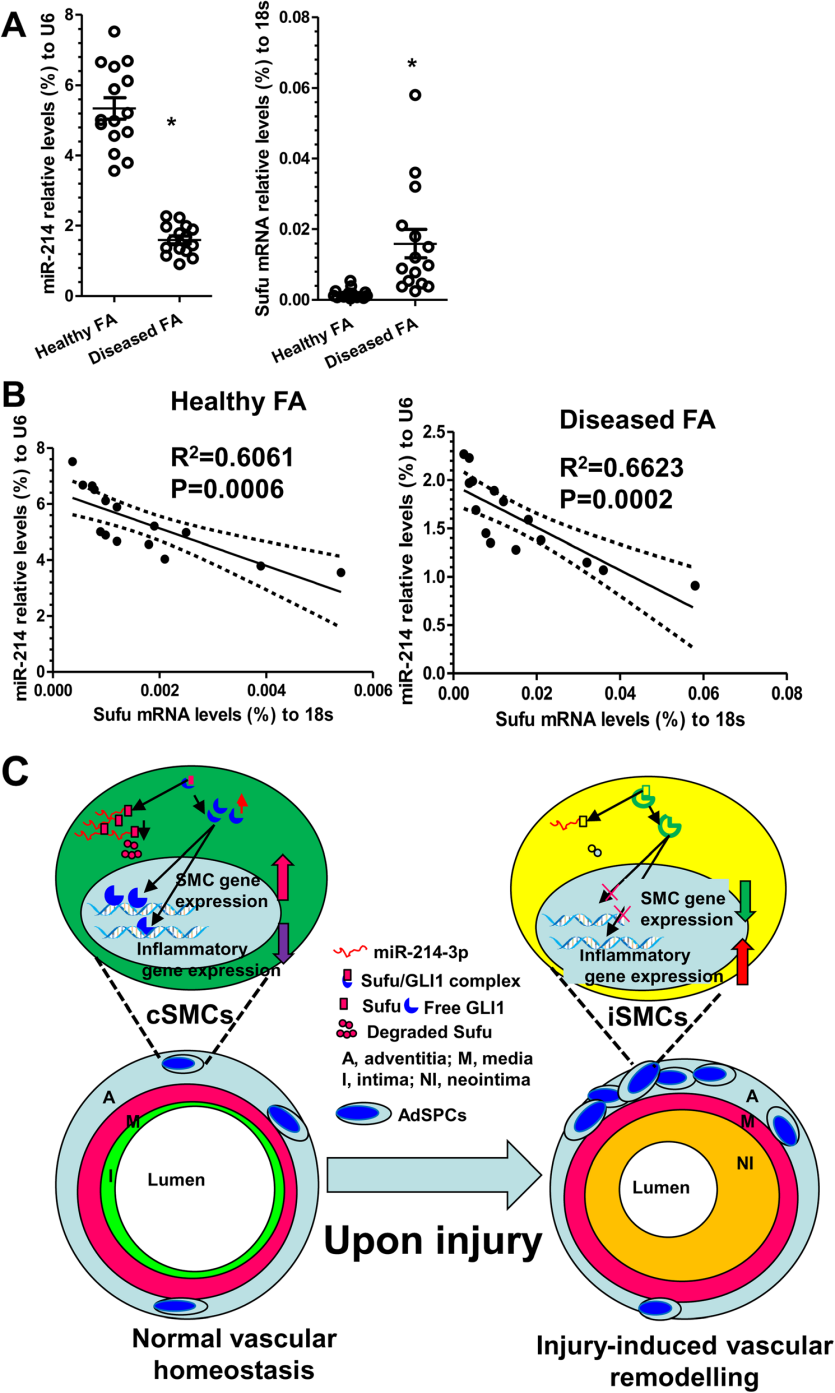
miR-214-3p and Sufu gene expression in human arteries. Healthy and diseased femoral artery (FA) specimens from patients without or with peripheral arterial diseases were collected and subjected to RT-qPCR analyses (*n* = 15 for each group). **a** miR-214-3p (relative to U6, %) and Sufu (relative to 18S, %) gene expression levels in each specimen (dots) were presented here. Bars indicate the mean ± S.E.M. in each cohort. *P < 0.05 (Mann-Whitney *U* test). **b** Spearman’s rank correlation analyses. The solid line indicates the fitted linear regression line; the dotted line indicates the 95% CI level. R is Spearman’s correlation coefficient between the expression levels of miR-214-3p and Sufu. *P* is the *P* value indicating the significant level of correlation. miR-214 indicates miR-214-3p. **c** Schematic illustration showing the model of action for the miR-214-3p/Sufu/GLI1 regulatory axis in iSMC differentiation from AdSPCs and injury-induced neointima formation. Under physiological condition, AdSPCs differentiate towards contractile SMCs (cSMCs), contributing to normal vascular homeostasis, whereas upon injury or inflammatory cytokine stimulation, increased AdSPCs differentiate into inflammatory SMCs (iSMCs), participating in injury-induced arterial remodelling. miR-214-3p plays a dural role in these processes. It promotes cSMC differentiation from AdSPCs under normal physiological condition but prevents iSMC generation from AdSPCs upon injury, thereby inhibiting the neointimal formation

To further validate and translate our findings into a clinical setting, the gene expression levels of miR-214-3p and Sufu and their possible relationship were examined in healthy and diseased femoral arterial (FA) specimens collected in our previous study [18]. RT-qPCR data showed decreased expression levels for the miR-214-3p gene, with an increased gene expression levels for Sufu in diseased femoral arteries, compared with healthy arteries (Fig. 7a). Importantly, we observed a significant inverse relationship between miR-214-3p and Sufu in both healthy and diseased femoral arterial specimens (Fig. 7b). Thus, the above human data supports the functional implication of miR-214-3p and its target gene Sufu in human angiographic restenosis.

## Discussion

The potential contributions of residential AdSPC-derived iSMCs to neointimal SMC hyperplasia and the underlying molecular mechanisms of iSMC differentiation/generation from the AdSPCs have yet to be fully recognised and elucidated and require further investigation. In the current study, we attempted to address this unexplored avenue by uncovering a novel role for miR-214-3p in modulating SMC differentiation and iSMC generation from AdSPCs in vitro and in vivo (Fig. 7c). Importantly, our data showed that miR-214-3p is highly upregulated during SMC differentiation from AdSPCs induced by TGFβ1 and provided clear evidence to suggest an important role for miR-214-3p in this process. Moreover, the addition of TNFα into the differentiating SMCs could prime them into an iSMC phenotype, a process which miR-214-3p over-expression could reverse. We have also demonstrated for the first time that the miR-214-3p-Sufu-GLI1 axis is one of the key signalling pathways responsible for iSMC differentiation/generation from AdSPCs. Mechanistically, we have confirmed that Sufu is an authentic downstream target of miR-214-3p during iSMC differentiation from AdSPCs and have identified Sufu as a novel transcriptional repressor of SMC contractile genes and an important transcriptional activator of inflammatory genes. Our ex vivo and in vivo data have shown for the first time that AdSPCs can be induced to differentiate into iSMCs within injured arteries and that transplanted AdSPCs over-expressing miR-214-3p onto injured arteries can reverse the process of iSMC differentiation from AdSPCs and prevent post-angioplasty restenosis. Importantly, our human data also support a potential role for miR-214-3p and Sufu in human atherosclerosis.

miR-214-3p belongs to the miR-199a-214 cluster, which is encoded by DNM3os that is transcribed from the opposite strand of the DNM3 gene [49]. Differing roles for miR-214-3p in cardiac cell functions and associated diseases have been well-documented in the literature. Reasons for the reported discrepancies are likely due to the fact that different cell systems and animal models were used among these studies, suggesting that miR-214-3p plays a divergent role in various diseases and the functional implication of miR-214-3p is cell context-dependent. A functional role for miR-214-3p in mature vascular SMCs and injury-induced arterial remodelling has been reported in our previous study. In that study, we reported that miR-214-3p controls both vascular SMC proliferation and migration, and miR-214-3p over-expression in the injured arteries reduces neointimal SMC hyperplasia by modulating vascular SMC proliferation [32]. In this study, we provide new perspectives on the role of miR-214-3p in iSMC generation from AdSPCs in the context of injury-induced arterial remodelling. Firstly, we explore the novel concept of AdSPCs as an additional cellular source for neointimal SMCs, while previously, we kept within the traditional confines of mature vascular SMC phenotypic modulation as the cause of neointimal SMC hyperplasia. Secondly, we have confirmed an additional function for miR-214-3p, namely that even in the presence of strong inflammatory stimuli, miR-214-3p can reverse the inflammatory phenotype of AdSPC-derived iSMCs into a contractile phenotype in vitro and in vivo. Thirdly, a novel underlying mechanism for iSMC differentiation/generation from AdSPCs has been documented in this study. Specifically, we demonstrate that Sufu is the true target gene of miR-214-3p in the context of iSMCs and injury-induced arterial remodelling. Sufu exerts its functions by allowing GLI1, the nuclear transcriptional effector of Shh signal pathway, to translocate into the nucleus to regulate relevant target gene expression. Finally, we are also able to translate our findings from mice into men by documenting the gene expression profiles of miR-214-3p and Sufu, as well as their relationships in both healthy and diseased human arteries. Therefore, findings from this study and previous ones support an important role of miR-214-3p in iSMC differentiation/generation from AdSPCs, vascular SMC biology and arterial remodelling induced by injury.

One of the new aspects of this study is the establishment of a reliable cellular model to study iSMC differentiation/generation from AdSPCs. Apart from the classical ‘contractile’ to ‘synthetic’ phenotypic modulation model, extensive evidence also suggests that vascular SMCs can be primed to another distinct phenotype, namely the ‘pro-inflammatory’ phenotype or iSMCs, in response to vascular injury or inflammatory stimuli. These iSMCs contribute to vascular disease progression and are a pervasive feature of atherosclerotic lesion formation [39] and arterial remodelling [50]. However, the majority of previous studies used mature vascular SMCs as a cellular model to study iSMCs and relevant signal pathways [51–53]. Although the existence and functional importance of stem/progenitor cell-derived SMCs in vascular diseases has been increasingly recognised by vascular biology researchers, there is a lack of cellular models with which to study AdSPC-derived iSMCs and the molecular mechanisms involved. In this study, we tested the hypothesis that exposure of the differentiating AdSPCs/SMCs with inflammatory stimuli could induce an iSMC phenotype from AdSPCs. Indeed, we found that inclusion of TNFα into the SMC differentiation medium could prime AdSPC-derived SMCs to an iSMC phenotype (Fig. 1 and S4), which is characterised by the decreased expression levels of SMC contractile genes and increased expression levels of inflammatory genes. As expected, compared to mature SMCs with different phenotypes, iSMCs produce and secrete the highest amount of inflammatory cytokines, IFNγ, IL-6 and MCP-1/CCL2. Interestingly, AdSPC-derived iSMCs also express high levels of S100A4, the reported marker for R-SMCs [40]. Although our iSMCs morphologically resemble R-SMCs [48], it would be difficult to claim that iSMCs are the same as or similar to R-SMCs, or equally represent a separate SMC phenotype, without a side-by-side comparison in terms of their functionalities, pathological contributions to vascular diseases and global gene/protein expression profiles.

Another new finding from this study is the identification of Sufu as a functional target gene of miR-214-3p in the context of iSMC differentiation from AdSPCs. Although multiple miR-214-3p target genes have been reported in various cellular contexts and diseases (e.g., NCKAP1 [32], N-RAS [28], ITCH [54], Quaking [26] and Ncx1), none of them seems to play a role in iSMC differentiation from AdSPCs. Instead, through our combined efforts, we have demonstrated that Sufu is a novel target gene of miR-214-3p in iSMCs. The Shh-GLI1 pathway has been reported as a key fate-determining factor for residential vascular stem cells [55]. Several components of the Shh-GLI1 signal pathway (e.g., Shh, patched-1, patched-2 and GLI1) have been used to mark the resident vascular SPCs [42, 56]. Importantly, Sufu is a well-known negative regulator of hedgehog signalling. It serves as a docking protein to retain the nuclear effector protein of Shh signalling, GLI1, in the cytoplasm, preventing it from being shuttled into the nuclei to trigger the transcriptional programme [45, 46], pointing to a possible signalling pathway for Sufu in vascular stem/progenitor cell development and differentiation. As it stands, this has yet to be reported in the current literature, and the mechanism through which Sufu (or Shh-GLI1 signal axis) is modulated in the context of SMC differentiation from AdSPCs remains elusive. In this study, we provide first-hand evidence to support that the Shh-GLI1 signal axis is activated during iSMC differentiation from AdSPCs, Sufu is negatively regulated by miR-214-3p and that the Shh-GLI1 signal axis is closely modulated by miR-214-3p/Sufu in iSMCs. NCKAP1 has previously been identified as a target gene of miR-214-3p in mature vascular SMCs [32]; however, we found no evidence to support the NCKAP1 as the functional target gene during iSMC differentiation from AdSPCs. These findings further support our understanding that any given miRNA can play divergent roles in response to different microenvironmental clues by regulating specific gene expression and that the way in which miRNAs regulate their target genes is dependent on the cellular context.

One of the novel mechanistic findings reported here is that Sufu could serve as a negative or positive regulator of SMC and inflammatory gene expression, respectively. We have demonstrated that Sufu is a new mediator to govern iSMC differentiation by regulating SMC-specific gene (SMαA and SM-myh11), SMC transcriptional factor SRF and inflammatory genes (IFNγ, IL-6 and MCP-1/CCL2) through a transcriptional mechanism. Our data shows that Sufu exerts transcriptional regulation through modulation of GLI1 nuclear translocation. Data generated from luciferase activity (Figs. 4c, f; 5e, f; and S6B, S7) and CHIP assays (Fig. 4g and S7G) provide multiple lines of evidence to support this mechanism. Specifically, our data shows that (1) Sufu negatively regulates SMC gene promoter activity (pGL3-SMαA, pGL3-SM22α and pGL3-SRF), (2) GLI1-binding sites within SMαA and SRF gene promoters are required for these gene transduction regulated by Sufu, (3) GLI1 nuclear accumulation and its binding to the promoter DNA of SMC-specific genes (SMαA and SRF) can be modulated by Sufu, and (4) the promoter activity of inflammatory genes (IL-6 and MCP-1/CCL2) is positively regulated by Sufu in iSMCs. The above-mentioned evidence clearly supports the claim that Sufu/GLI1 may function as a transcriptional activator or repressor for distinct sets of genes in response to the micro-environmental clues. Similarly, we have also noticed that S100A4 gene expression is closely modulated by the miR-214-3p/Sufu axis during iSMC differentiation from AdSPCs. Therefore, it would be very interesting to investigate whether Sufu regulates S100A4 gene expression through a similar mechanism.

## Conclusions

In the current study, we have successfully uncovered the functional involvements of miR-214-3p in iSMC differentiation/generation from residential AdSPCs in the context of vascular remodelling after injury (Fig. 7c). Moreover, the newly identified target gene Sufu is at least partially responsible for miR-214-3p-mediated iSMC differentiation/generation from AdSPCs. Therefore, the findings presented in this study suggest that modulating miR-214-3p-Sufu-GLI1 axis could be a potential therapeutic tool for vascular diseases such as atherosclerosis and post-angioplasty restenosis.

## Supplementary Information

**Additional file 1: Table S1 and Supporting Methods.**

**Additional file 2: Figure S1.** Characterization of adventitia stem/progenitor cells (AdSPCs). (A) Phase-contrast image showing the primary culture of AdSPCs (P0). (B)AdSPCs at passage 3~8 were subjected to immunofluorescence staining with antibodies against AdSPC and other cell lineage markers, namely Sox10/FSP1 (fibroblast marker), Sox17/CD31 (endothelial cell marker), and Nestin/SM-NHC (SMC marker), respectively. (C) Flow cytometry analysis of AdSPC markers (Sox10 and Nestin) expression in AdSPCs at passage 3. (D) Gene expression profiles in AdSPCs at the indicated passage number (P). The data presented here are representative (A-C) or mean±S.E.M. (D) of five independent experiments. *P<0.05 (versus P3, one-way ANOVA with a post hoc test of Tukey’s analysis).

**Additional file 3: Figure S2.** SMC differentiation from AdSPCs. Passage 3-8 AdSPCs were cultured in SMC differentiation medium (DMEM supplemented with 5% FBS and 5ng/ml TGFβ1) for the indicated time to allow for SMC differentiation. Total RNAs and proteins from undifferentiated AdSPCs (0d) or differentiating AdSPCs at day 2, 4, 6 and 8 were harvested and subjected to RT-qPCR (A) and Western blot (B) analyses, respectively. (C) Mature SMC marker expression in AdSPC-derived SMCs. Day 8 differentiated SMCs were stained with antibodies against SMαA and SM-MHC. The data presented here are representative images (left panel in B, and C) or mean±S.E.M. (A, and right panel in B) of five independent experiments. *P<0.05 (versus 0d, one-way ANOVA with a post hoc test of Tukey’s analysis).

**Additional file 4: Figure S3.** miR-214 promotes SMC differentiation from AdSPCs. (A) Increased expression of miR-214 in AdSPCs in response to TGFβ1. (B-E) miR-214 modulates SMC marker expressions. Day 2 differentiating AdSPCs were transfected with miR-214 mimics, inhibitor or respective negative control (miR ctrl), and cultured for 2~3 days, followed by RT-qPCR (B & C) and western blot (D & E) analyses, respectively. The data presented here are representative (up panels in D & E) or mean±S.E.M. (A-C, and bottom panels in D & E) of five independent experiments. *P<0.05. One-way ANOVA with a post hoc test of Tukey’s analysis for A & C, unpaired t-test for B. miR-214 indicates miR-214-3p.

**Additional file 5: Figure S4.** Characterisation of iSMC phenotype. Mature SMCs were subjected to serum starvation for 48 hours, followed by the indicated treatments for additional 24 hours. Total RNAs and conditioned culture medium harvested from SMCs treated with vehicle (serum-starved SMCs, ssSMCs), 5ng/ml TGFβ1 (‘contractile’ SMCs, conSMCs), 10ng/ml PDGF-BB (‘synthetic’ SMCs, synSMCs), and iSMCs differentiated from AdSPCs (iSMCs) were subjected to RT-qPCR (A) and ELISA analyses (B), respectively. (C) Luciferase activity assays detected NFκB activation in cells with the indicated treatments. (D) BrdU incorporation assays to detect cell proliferation. (E) Trans-well migration assays detected cell migration under 30ng/ml PDGF-BB stimulation. The data presented here are representative images (left panels in E) or mean±S.E.M. (A-D, right panel in E) of five independent experiments (*n*=5). *P<0.05 (versus ssSMCs), ^#^<0.05 (versus synSMCs) (one-way ANOVA with a post hoc test of Tukey’s analysis).

**Additional file 6: Figure S5.** GLI1 cellular location was modulated by miR-214-3p. AdSPCs were differentiated into iSMCs (DMEM containing 5ng/ml TGFβ1 and 50ng/ml TNFα) for 4 days, then transfected with miR-214-3p mimics (miR-214) or negative control (miR ctrl), and cultured for 48 hours in the same culture medium. Cells were fixed and subjected to immunofluorescence staining (A). Fifty cells were randomly selected for quantification by Image J free software (Version 1.47, RRID:SCR_003070) from each treatment. Mean fluorescence intensity (MFI) of nuclear (B) and cytoplasmic (C) GLI1 (green), as well as DAPI (blue) from the same cells were quantified, and the ratio of MFI of GLI1 over DAPI were calculated accordingly. Veh indicates vehicle control for TNFα. The data presented here are representative (A) or mean±S.E.M. (B & C) of fifty cells (*n*=50). *P<0.05 (vs veh/miR ctrl); ^#^P<0.05 (TNFα/miR-214 vs TNFα/miR ctrl) (one-way ANOVA with a post hoc test of Tukey’s analysis). miR-214 indicates miR-214-3p.

**Additional file 7: Figure S6.** Sufu controls SMC gene expression and GLI1 cellular location. (A) SMC gene expression is negatively regulated by Sufu. AdSPCs (p3~p8) were infected with control (pCDH) or Sufu over-expression (pCDH-Sufu) lentivirus and cultured in iSMC differentiation induction medium for 2~3 days. The expression levels of a panel of SMC specific genes was examined by RT-qPCR analysis. (B) Sufu over-expression inhibited SMC gene promoter activity. AdSPCs infected with pCDH or pCDH-Sufu were transfected with SMC gene promoter reporters (pGL3-SMαA or pGL3-SM22α) as indicated. Luciferase activity assays were conducted at 48 hours post-transfection. (C) GLI1 gene expression was not affected by Sufu over-expression. (D) Sufu knockdown promotes GLI1 protein accumulation in nuclei. AdSPCs were infected with non-target (sh-NT) or Sufu (sh-Sufu) shRNA lentivirus, followed by iSMC differentiation. Cells were fixed and subjected to immunofluorescence staining with GLI1 antibody. Data presented here are representative images (D) or Mean±S.E.M of five or six independent experiments (*n*=5 or 6, unpaired t-test).

**Additional file 8: Figure S7.** Sufu controls SMC gene expression through regulating SRF. (A-B) Serum response factor (SRF) binding sites within SMC gene promoters are required for Sufu-mediated SMC gene repression. AdSPCs infected with non-target (sh-NT) or Sufu (sh-Sufu) shRNA lentivirus were transfected with wild type (WT) SMC gene promoter reporters (pGL3-SMαA or pGL3-SM22α), or SRF binding site mutants [SRF^mut^], respectively. Luciferase activity assay were measured at 48 hours post-transfection. (C-D) Sufu over-expression inhibited SRF gene expression, while Sufu knockdown promoted its expression. (E) SRF gene promoter activity is increased by Sufu inhibition. (F) GLI1 binding site within SRF gene promoter region is necessary for SRF gene up-regulation by Sufu knockdown in AdSPCs. The potential wild type binding site (pGL3-SRF-WT) of GLI1 within SRF gene promoter and its mutant pGL3-SRF-GLI1^mut^) are depicted in this illustration (Insert). AdSPCs infected with sh-NT or sh-Sufu lentivirus were transfected with respective SRF gene promoter reporters as indicated. Luciferase activity assay were measured at 48 hours post-transfection. (G) Sufu inhibition increases GLI1 enrichment within SRF gene promoter. ChIP assays were performed using antibody against GLI1 or normal IgG, respectively. PCR amplifications of the adjacent regions were included as additional control for specific promoter DNA enrichment. Data presented here are Mean±S.E.M of five or six independent experiments (*n*=5 or 6, unpaired t-test).

**Additional file 9:** **Figure S8.** miR-214 decreases iSMCs during arterial remodelling. After wire-induced femoral arterial injury, 100 μl of 30% pluronic gel containing 2.5 nmol control AgomiR (Cel-miR-67) or miR-214 AgomiR (miR-214) per artery per mouse was immediately applied and packed around the injured femoral arteries. Two weeks later, injured segments of femoral arteries were harvested for H&E staining analyses. Mice underwent a sham arterial injury procedure serve as control (Sham). SMCs and neointima (NI) cells were isolated from media of sham mice (SMCs/Sham) or media/neointima layers of injured mice received control (NI cells/Cel-miR-67) or miR-214 (NI cells/miR-214) AgomiR, respectively. Isolated cells were cultured in SMC medium for 5-7 days and subjected to various analyses as indicated. (B) Phase-contrast images were also taken prior to other analyses and included here. (C & D) Immunofluorescence staining assays with antibodies against SMαA and IL-6. Representative images (C) and percentage of iSMCs from each group ((D) were presented here. (E) RT-qPCR detection of gene expression. Data presented here are representative images or Mean±S.E.M of five independent experiments (n=5). *P<0.05 (versus sham), ^#^P<0.05 (miR-214 versus Cel-miR-67) (one-way ANOVA with a post hoc test of Tukey’s analysis). miR-214 indicates miR-214-3p.

**Additional file 10: Figure S9.** Transplanted AdSPCs differentiate toward iSMCs during arterial remodelling. After injury, 100μl of Geltrex contained 1x10^6^ AdSPCs infected with Lenti-GFP per vessel per mice was immediately applied and packed around injured vessel. One week later, injured segments of femoral arteries were harvested and prepared, followed by triple immunofluorescence staining with antibodies against GFP, SMαA and S100A4. White arrows within merged image indicate iSMCs derived from transplanted AdSPCs. Data presented here are representative images from three mice.

## Data Availability

Some data/material may not be made available because of privacy or ethical restrictions. All remaining data are contained within the article.

## Abbreviations

miR-214-3p: MicroRNA-214-3p
Sufu: Suppressor of fused
SRF: Serum response factor
Shh: Sonic hedgehog
GLI1: Glioma-associated oncogene 1
SMC: Smooth muscle cell
iSMC: Inflammatory smooth muscle cell
R-SMC: Rhomboid SMC
AdSPCs: Adventitial stem/progenitor cells
TGFβ1: Transforming growth factor beta1
TNFα: Tumour necrosis factor alpha
IFNγ: Interferon gamma
MCP-1: Monocyte chemoattractant protein 1
SMαA: Smooth muscle α actin
SM22α: Smooth muscle 22α
SM-myh11: Smooth muscle myosin heavy chain 11
SM-MHC: Smooth muscle myosin heavy chain
S100A4: S100 calcium-binding protein A4
FSP1: Fibroblast-specific protein 1
Sox10 or 17: SRY-box transcription factor 10 or 17
3′UTR: 3′-Untranslated region
BS: Binding site
shRNA: Short hairpin RNA
ChIP: Chromatin immunoprecipitation
GFP: Green fluorescent protein
RT-qPCR: Real-time quantitative PCR
Cel-miR-67 AgomiR: *Caenorhabditis elegans* miR-67 AgomiR
FA: Femoral artery
NI: Neointima
