# Supplementary material for: miR-214-3p-Sufu-GLI1 is a novel regulatory axis controlling inflammatory smooth muscle cell differentiation from stem cells and neointimal hyperplasia
Source: Stem Cell Res Ther. 2020 Nov 3;11:465. doi: 10.1186/s13287-020-01989-w (PMC7640405; doi:10.1186/s13287-020-01989-w)
Supplement: Supplementary file 1 — Additional file 1: Table S1 and Supporting Methods. [file 13287_2020_1989_MOESM1_ESM.pdf]

Table S1: Primer sets used in the present study

Gene names	Forward (5’-3’)	Reverse (5’-3’)	Application
U6 snoRNA (mus/hu)	gatgacacgcaaattcgtg	miRNA universal reverse primer (Invitrogen, A11193-051)	RT-qPCR
miR-214 (mus/hu)	CAGGCACAGACAGGCAGT	miRNA universal reverse primer (Invitrogen, A11193-051)	RT-qPCR
miR-34a (mus/hu)	GGCAGTGTCTTAGCTGGTTGT	miRNA universal reverse primer (Invitrogen, A11193-051)	RT-qPCR
miR-22 (mus/hu)	AAGCTGCCAGTTGAAGAACTGT	miRNA universal reverse primer (Invitrogen, A11193-051)	RT-qPCR
18s (mus/hu)	AAACGGCTACCACATCCAAG	CCTCCAATGGATCCTCGTTA	RT-qPCR
Mus SMαA	TCCTGACGCTGAAGTATCCGAT	GGCCACACGAAGCTCGTTATAG	RT-qPCR
Mus SM22α	GATATGGCAGCAGTGCAGAG	AGTTGGCTGTCTGTGAAGTC	RT-qPCR
Mus CNN1	GCGTCACCTCTATGATCCCAA	CCCAGACCTGGCTCAAAGAT	RT-qPCR
Mus SM-myh11	AAGCAGCCAGCATCAAGGAG	AGCTCTGCCATGTCCTCCAC	RT-qPCR
Mus SRF	CCTACCAGGTGTCGGAATCTGA	TCTGGATTGTGGAGGTGGTACC	RT-qPCR
Mus IFNγ	CGGCACAGTCATTGAAAGCC	TGTCACCATCCTTTTGCCAGT	RT-qPCR
Mus IL-6	GTGGCTAAGGACCAAGACCA	TAACGCACTAGGTTTGCCGA	RT-qPCR
Mus MCP-1/CCL2	CCCCAAGAAGGAATGGGTCC	TGCTTGAGGTGGTTGTGGAA	RT-qPCR
Mus S100A4	TCCTCTCTCTTGGTCTGGTCT	GTCACCCTCTTTGCCTGAGT	RT-qPCR
Mus Shh	TCTCGAGACCCAACTCCGAT	GACTTGTCTCCGATCCCCAC	RT-qPCR
Mus Ptch1	CCAGCGGCTACCTACTGATG	TGCCAATCAAGGAGCAGAGG	RT-qPCR
Mus GLI1	GTCTGCGTGGTAGAGGGAAC	GGAGACAGCATGGCTCACTA	RT-qPCR
Mus Wnt1	AACAGTAGTGGCCGATGGTG	CCAGCTGCAGACTCTTGGAA	RT-qPCR
Mus Wnt4	ATCTCTTCAGCAGGTGTGGC	TGTTGTCCGAGCATCCTGAC	RT-qPCR
Mus Wnt9a	CGGGGACAACCTCAAGTACA	ATCACCTTCACACCCACGAG	RT-qPCR
Mus Wisp1	CACAGATGGCTGTGAATGCTG	ACCTGTGCACACACTCCTAT	RT-qPCR
Mus Sufu	ACCTGCAACAGGAGAGAGTT	GATGCTCCGGCTATCCTCTTC	RT-qPCR
Hu Sufu	CCACACCTGCAAGAGAGAGT	TTGGCACTGACACCACTCAG	RT-qPCR
Mus Col1a1	AGCACGTCTGGTTTGGAGAG	GACATTAGGCGCAGGAAGGT	RT-qPCR
Mus ECM1	CTCTTCCAGCACCTCCATGA	GGGAGGGTCATGCAGAGAAG	RT-qPCR
SMαA promoter-1	CATAACGAGCTGAGCTGCCTC	CCAAACAAGGAGCAAAGACG	CHIP assay
SMαA promoter-2	GATCAGAGCAAGGGGCTATA	CTACTTACCCTGACAGCGAC	CHIP assay
SMαA adjacent	GGCAACACAGGCTGGTTAAT	AGCCCCTGTCAGGCTAGTCT	CHIP assay
SRF promoter	TCAGGCCTGTGCTTTAGCCTCG	GATGGGGGCAGGGCGGAAAG	CHIP assay
SRF adjacent	CCTGGCTGGCTTGGCACTCAC	ATCTGGCCGGACGGTGTGATA	CHIP assay
pGL3-SMαA-GLI1^{mut-1}	GAGACCCAGCCTCT GGTTATTTA GATTAGAGAGTTTTGTGC	GCACAAAACTCTCTAATCTAAATAA CCAGAGGCTGGGTCTC	GLI1 binding site-1 mutation
pGL3-SMαA-GLI1^{mut-2}	CAGCCTCCCGGGACATTATTTACCC AGAGTGGAGAAGCC	GGCTTCTCCACTTGGGTAAATAATG TCCCGGGAGGCTG	GLI1 binding site-2 mutation
pGL3-SRF-CLI1^{mut}	GTGCTTTAGCCTCGAGATTATTTC CCACCCCCCTTTTAGCAC	GTGCTAAAAGGGGGGTGGGAAATA ATCTCGAGGCTAAAGCAC	GLI1 binding site mutation
pmiR-Luc-EVI1-WT	CTCCTC GAGCTC TCAATTTGTCTGGCCTGGGG	GAGGAG AAGCTT GTAGCCCCAGGAACTGAGTC	Mus Sufu 3’UTR reporter clone
pmiR-Luc-EVI1-BS^{mu}	ctcaggaatg tctaaactcACGCGTggacc agaccctgtc	gacagggtct ggtcc ACGCGTgagtttaga cattcctgag	miR-214 binding site mutation

Detailed Materials and Methods

1. Materials

Antibodies against Smooth Muscle Myosin Heavy Chain 11 (SM-MHC) (rabbit IgG, ab53219, RRID:AB_2147146), Sox10 (mouse IgG, ab216020, RRID:AB_2847913), GLI1 (rabbit IgG, ab49314, RRID:AB_880198), Lamin B1 (rabbit IgG, ab133741, RRID:AB_2616597), Sufu (rabbit IgG, ab28083, RRID:AB_2255467), S100A4 (rabbit IgG, ab197896, RRID:AB_2728774), IL-6 (rabbit IgG, ab7737, RRID:AB_306031), and GFP (chicken IgG, ab13970, RRID:AB_300798) were purchased from Abcam, UK. Antibodies against α-tubulin (mouse IgG, T6074, RRID:AB_477582), and monoclonal anti-α smooth muscle actin (SMαA) (Clone 1A4, mouse IgG, A5228, RRID:AB_262054) were from Sigma. All secondary antibodies and other materials were from Sigma or ThermoFisher Scientific UK unless specifically indicated. All the growth factors or cytokines were purchased from form Biolegend UK unless specifically indicated.

2. AdSPC isolation, culture and identification

The procedures for mouse aortic AdSPC isolation and culture were described in our previous study(1). Briefly, aortas were carefully harvested from 8 to 10-week old mice and incubated in collagenase I for 15 min. Under a dissecting microscope adventitia were carefully dissected and cut into 1-2 mm size. Tissue blocks from 10 mice were pooled together and digested in 3 mg/ml type II collagenase in DMEM for 30 min. After then the same volume of 1 mg/ml elastase solution was added to the digest solution and incubated for another 1–2 h until all the tissues were digested. After filtering with a Cell Strainer (70μm), single cell digestion solution was centrifuged to remove the digestion solution. Cells were re-suspended with AdSPC maintenance medium (DMEM with 2% chick embryo extract, 1% FBS, 1% N2, 2% B27, 100 nM retinoic acid, 50 nM 2-mercaptoethanol, 1% P/S and 20 ng/ml bFGF) and transferred to six-well culture plate pre-coated with 1% CellStart (Invitrogen, A1014201). AdSPCs were maintained in the AdSPC maintenance medium for up to 10 passages. Every batch of AdSPCs at passage 3 was tested by AdSPC marker Sox10 staining to ensure the purity of primary AdSPCs above 95%.

3. Sufu shRNA lentiviral construction and infection

As described in our previous study(2), the non-target and Sufu specific shRNA lentiviral particles were produced using the shRNA Non-Targeting control vector (SHC002) and MISSION shRNA Sufu plasmids DNA (SHCLNG- NM_015752, Sigma), respectively, according to protocol provided. For lentiviral infection, one transducing Unit per cell (or 2-3x10^6/well) of lentivirus was introduced into AdSPCs with 10μg/ml hexadimethrine bromide (H9268; Sigma). At 40~48 hours post viral infection, the cell culture medium was replaced with complete media containing 2μg/ml puromycin (P9620, Sigma), for transductants selection. Stably infected cells were split and frozen for future experiments.

4. Generation of Sufu over-expression pseudoviral particles

The procedures for constructing Sufu over-expression pseudoviral particles were similar to the protocols described in our previous studies(3,4). The GenEZ ORF Clone (Clone ID: OMu12159C) for mouse Sufu DNA (NM_001025391) was purchased from GeneScript, and subcloned into the NheI and NotI sites of pCDH-CMV-MCS-

EF1-Puro vector (CD510B-1, System Biosciences), designated as pCDH-Sufu. Control vector pCDH-GFP was generated in our previous studies(3,4). All vectors were verified by DNA sequencing. Control (pCDH or Lenti-GFP) and Sufu (pCDH-Sufu) pseudoviral particles were generated using pCDH, pCDH-GFP, or pCDH-Sufu and packaging plasmids, respectively, as described in our previous studies(3,4).

5. SMC gene promoters and GLI mutants

SMC gene promoters (pGL3-SMαA/SM22α-WT)(5) and their corresponding serum response factor (SRF) binding site mutants (pGL3-SMαA/SM22α-SRF^{mut})(6) were generated in our previous studies. Similarly, SRF gene prompter (pGL3-SRF) was also generated in our previous study(6). GLI binding site mutation(s) were introduced into the respective pGL3-SMαA-WT or pGL3-SRF-WT plasmids by using QuikChange™ site-directed mutagenesis kit (Agilent Technologies) according to the manufacturer's instructions, and designated as pGL3-SMαA/SRF-GLI1^{mut}, respectively. All vectors were verified by DNA sequencing.

6. Sufu 3'UTR clone and miR-214-3p binding site mutation. Reporter vector harboring sequences of the murine Sufu 3'UTR was created using cDNA from AdSPCs. The fragment containing 3'-flanking untranslation region (3'UTR) of murine Sufu gene (NM_001025391) was amplified by PCR with primer sets shown in **Online table S1** and cloned into the KpnI and XbaI sites of the pmiR-reporter-basic vector (Ambion, Applied Biosystems), designated as pmiR-Luc-Sufu-WT. miR-214-3p binding site mutation was introduced into this reporter by using QuikChange™ site-directed mutagenesis kit (Agilent Technologies) according to the manufacturer's instructions, and designated as pmiR-Luc-Sufu-miR-214-BS^{mut}. All vectors were verified by DNA sequencing.

7. Plasmid transient transfection and luciferase assay

Luciferase assays for gene promoter activity were conducted as with previous studies(7,8).

For Shh-Gli1 signalling reporter assay, AdSPCs were transfected with GLI/Renilla mixed reporter plasmids (40:1) (Qiagen, CCS-6030L) using TurboFect Transfection Reagent (Thermo Fisher Scientific Inc.) according to the manufacturer's instructions, followed by various treatments as indicated in the respective figure legend.

For determining the respective gene promoter activity, the individual promoter vectors ((pGL3-SMαA-WT, pGL3-SM22α-WT, pGL3-SMαA-SRF^{mut}, pGL3-SM22α-SRF^{mut}, pGL3-SMαA-GLI1$^{mut-1, -2, or -1/2}$, pGL3-SRF-WT, or pGL3-SRF-GLI1^{mut}) (0.15 μg/2.5 x 10^4 cells) for SMC marker genes, pmIL-6 FL (Addgene, Plasmid, #61286, a kind gift from Gail Bishop(9)) for murine IL-6 gene, or pMCP-1 (Addgene, Plasmid, #40324, a kind gift from Alexander Dent(10)) for murine MCP-1/CCL2 gene) (0.25 μg/2.5 x 10^4 cells) were transfected into differentiating AdSPCs with various treatments as indicated in the respective figure legend. Additionally, the reporter vector containing five copies of an NF-κB response element (NF-κB-RE) (pGL4-NF-κB or pGL4.32[luc2P/NF-κB-RE/Hygro] was used for measuring NF-κB signalling, Promega, E8491).

Apart from Shh-Gli1 signalling reporter assay, p*Renilla* (15 ng/2.5 x 10^4 cells) was included in all transfection assays as internal control. Dual-Luciferase Reporter Assay

System was used for detecting luciferase and *Renilla* activities according to the protocol provided in the system. Relative luciferase unit (RLU) was defined as the ratio of Luciferase versus *Renilla* activity with that of the control (set as 1.0).

8. miR-214-3p mimics and inhibitor transfection

miR-214-3p mimics (mirVana® miRNA mimic, Catalog #4464066/MC12124) and inhibitor (mirVana® miRNA inhibitor, Catalog #4464084/MH12124) were purchased from ThermoFisher Scientific, UK. As described in our previous studies(11,12), both miRNA mimics and inhibitor (25 nmol/L, final concentration) were transfected into AdSPCs using TransIT-X2 transfection reagent (Geneflow Ltd), according to the manufacturer's instructions.

9. Real time quantitative PCR (RT-qPCR) for mRNA and microRNAs

Established and standardised real-time quantitative PCR (RT-qPCR) protocols to examine mRNA and microRNA expression were performed as previously described(7,8). Relative mRNA or microRNA expression level was defined as the ratio of target gene expression level or microRNA expression level to 18S or U6 snRNA expression level, respectively, with that of the control sample set as 1.0. In some experiments, relative mRNA or microRNA expression level was defined as the ratio of target gene expression level or microRNA expression level to 18S or U6 snRNA expression level, respectively. Primers were designed using Primer Express software (ThermoFisher Scientific Inc. UK) and the sequence for each primer was listed in **Online table S1**.

10. Immunoblotting

The whole cell lysate or the nuclear protein fractions (in some experiments) were generated using a Nuclear Extraction Kit from Abcam (ab113474) according to the provided protocol, and subjected to standard immunoblotting analysis. All the blots were subjected to densitometric analysis with Image J free software (Version 1.47, RRID:SCR_003070). Relative protein expression level was defined as the ratio of target protein expression to their corresponding loading control (α-tubulin), with that of the respective control sample set as 1.0.

11. Indirect immunofluorescent staining for cells. Indirect immunofluorescent assay was performed as previously described(7,8). Cells were labelled with isotype IgG control or the indicated primary antibodies, and visualized using appropriate secondary antibodies conjugated with Alexa Fluor Plus 647 or 488. Cells were counterstained with 4', 6-diamidino-2-phenylindole (DAPI; Sigma) and mounted in Fluoromount-G (Cytomation; DAKO, Glostrup, Denmark). Images were examined using EVOS FL Auto Cell Imaging System with Plan-NEOFLUAR 10x, 20x, or 40x objective lenses and provided software (ThermoFisher Scientific Inc. UK) at room temperature, and were processed with Photoshop software (Adobe).

12. ELISA analysis of S100A4 and other cytokines

S100A4 was assessed using a murine s100a4 ELISA Kit (ABIN4972603, Detection Limit: 15.6pg/ml) from Antibody online, while the levels of other cytokines in the conditioned culture medium were measured using their respective ELISA kit (EM2IL6 for IL-6 (7pg/ml); EMMCP1 for MCP-1 (4pg/ml); and KMC4021 for IFNγ

(<2pg/ml)) purchased from ThermoFisher, according to the manufacturer's instructions.

13. Chromatin immunoprecipitation (ChIP) assays. The ChIP assays were performed as previously described(7,8). Briefly, AdSPCs infected with control (sh-NT) or Sufu (sh-Sufu) shRNA lentivirus were induced to differentiate toward iSMCs. Differentiating cells were harvested and sonicated, and the sheared samples were immunoprecipitated with antibodies raised against GLI1 (rabbit IgG, LS-C150725-LSP, Stratech Scientific Ltd, UK), or rabbit IgG control (5µg/immunoprecipitation). Immunoprecipitation complex was pulled-down using protein-G Dynabeads, and the purified immunoprecipitated DNA was amplified by RT-qPCR using specific primers (**Online Table S1**) to detect the target DNA sequences. Promoter DNA enrichment with specific antibody was calculated using percent input method with that of the IgG control set as 1.0. PCR amplification of the adjacent promoter regions were included as additional control for specific promoter DNA enrichment. The data was obtained from five to six independent experiments.

14. Animal experiments, anaesthesia and euthanasia

All animal experiments were conducted according to the Animals (Scientific Procedures) Act of 1986 (United Kingdom). All the animal procedures were approved by Queen Mary University of London ethics review board (PPL number: PB508B78D), and conform to the guidelines from Directive 2010/63/EU of the European Parliament on the protection of animals used for scientific purposes or the NIH guidelines (Guide for the care and use of laboratory animals). For mouse carotid artery denudation injury and macrophage transplantation, anaesthesia was induced using 100% O_2/4% isoflurane, and was maintained throughout the procedure by the administration of 100% O_2/2% isoflurane. At the end of protocol, all mice were euthanized by placing them under deep anaesthesia with 100% O_2/5% isoflurane, followed by decapitation.

15. Morphometric analysis, quantification of lesion formation, and tissue immunofluorescence staining

The femoral arteries (~5.0mm from injury site) were harvested at 4 weeks post-injury, fixed in 4% formaldehyde, and proceeded for paraffin sectioning. Sections (5~6 µm) were collected at 200µm intervals (10 sections per segment/interval), mounted on slides, and numbered, followed by H&E staining for morphometric analysis and lesion quantification as described in our previous studies(11-13).

For immunofluorescence staining, the injured carotid arteries were collected at 7 days post-injury, and proceeded as described above. Three digitised sections with same identification number from three segments/intervals of each animal were subjected to standard immunofluorescence staining with indicated primary antibodies or respective IgG controls. The tissue sections were then washed and subsequently incubated with an appropriate Alexa Fluor Plus 488/594/647-conjugated 2nd antibody (ThermoFisher UK, 1:1000 dilution), followed by nuclei staining with 4,6-diamidino-2-phenylindole (1ug/ml). Laser scanning confocal microscope (Zeiss LSM 510 Mark 4) and Zen 2009 image software was used for image capture and inspection.

Supplementary Reference:

1. Yang, F., Chen, Q., Yang, M., Maguire, E. M., Yu, X., He, S., Xiao, R., Wang, C. S., An, W., Wu, W., Zhou, Y., Xiao, Q., and Zhang, L. (2020) Macrophage-derived MMP-8 determines smooth muscle cell differentiation from adventitia stem/progenitor cells and promotes neointima hyperplasia. *Cardiovascular research* **116**, 211-225
2. Xiao, Q., Zhang, F., Lin, L., Fang, C., Wen, G., Tsai, T. N., Pu, X., Sims, D., Zhang, Z., Yin, X., Thomaszewski, B., Schmidt, B., Mayr, M., Suzuki, K., Xu, Q., and Ye, S. (2013) Functional role of matrix metalloproteinase-8 in stem/progenitor cell migration and their recruitment into atherosclerotic lesions. *Circ Res* **112**, 35-47
3. Zhang, C., Chen, D., Maguire, E. M., He, S., Chen, J., An, W., Yang, M., Afzal, T. A., Luong, L. A., Zhang, L., Lei, H., Wu, Q., and Xiao, Q. (2018) Cbx3 inhibits vascular smooth muscle cell proliferation, migration, and neointima formation. *Cardiovascular research* **114**, 443-455
4. Zhang, L., Chen, Q., An, W., Yang, F., Maguire, E. M., Chen, D., Zhang, C., Wen, G., Yang, M., Dai, B., Luong, L. A., Zhu, J., Xu, Q., and Xiao, Q. (2017) Novel Pathological Role of hnRNPA1 (Heterogeneous Nuclear Ribonucleoprotein A1) in Vascular Smooth Muscle Cell Function and Neointima Hyperplasia. *Arterioscler Thromb Vasc Biol* **37**, 2182-2194
5. Margariti, A., Xiao, Q., Zampetaki, A., Zhang, Z., Li, H., Martin, D., Hu, Y., Zeng, L., and Xu, Q. (2009) Splicing of HDAC7 modulates the SRF-myocardin complex during stem-cell differentiation towards smooth muscle cells. *J Cell Sci* **122**, 460-470
6. Huang, Y., Lin, L., Yu, X., Wen, G., Pu, X., Zhao, H., Fang, C., Zhu, J., Ye, S., Zhang, L., and Xiao, Q. (2013) Functional involvements of heterogeneous nuclear ribonucleoprotein A1 in smooth muscle differentiation from stem cells in vitro and in vivo. *Stem Cells* **31**, 906-917
7. Zhao, H., Wen, G., Huang, Y., Yu, X., Chen, Q., Afzal, T. A., Luong le, A., Zhu, J., Ye, S., Zhang, L., and Xiao, Q. (2015) MicroRNA-22 regulates smooth muscle cell differentiation from stem cells by targeting methyl CpG-binding protein 2. *Arterioscler Thromb Vasc Biol* **35**, 918-929
8. Yu, X., Zhang, L., Wen, G., Zhao, H., Luong, L. A., Chen, Q., Huang, Y., Zhu, J., Ye, S., Xu, Q., Wang, W., and Xiao, Q. (2015) Upregulated sirtuin 1 by miRNA-34a is required for smooth muscle cell differentiation from pluripotent stem cells. *Cell Death Differ* **22**, 1170-1180
9. Baccam, M., Woo, S. Y., Vinson, C., and Bishop, G. A. (2003) CD40-mediated transcriptional regulation of the IL-6 gene in B lymphocytes: involvement of NF-kappa B, AP-1, and C/EBP. *J Immunol* **170**, 3099-3108
10. Toney, L. M., Cattoretti, G., Graf, J. A., Merghoub, T., Pandolfi, P. P., Dalla-Favera, R., Ye, B. H., and Dent, A. L. (2000) BCL-6 regulates chemokine gene transcription in macrophages. *Nat Immunol* **1**, 214-220
11. Afzal, T. A., Luong, L. A., Chen, D., Zhang, C., Yang, F., Chen, Q., An, W., Wilkes, E., Yashiro, K., Cutillas, P. R., Zhang, L., and Xiao, Q. (2016) NCK Associated Protein 1 Modulated by miRNA-214 Determines Vascular Smooth Muscle Cell Migration, Proliferation, and Neointima Hyperplasia. *Journal of the American Heart Association* **5**
12. Yang, F., Chen, Q., He, S., Yang, M., Maguire, E. M., An, W., Afzal, T. A., Luong, L. A., Zhang, L., and Xiao, Q. (2018) miR-22 Is a Novel Mediator of

Vascular Smooth Muscle Cell Phenotypic Modulation and Neointima Formation. *Circulation* **137**, 1824-1841

13. Chen, Q., Yang, F., Guo, M., Wen, G., Zhang, C., Luong le, A., Zhu, J., Xiao, Q., and Zhang, L. (2015) miRNA-34a reduces neointima formation through inhibiting smooth muscle cell proliferation and migration. *J Mol Cell Cardiol* **89**, 75-86
