# Supplementary material for: miR-214-3p-Sufu-GLI1 is a novel regulatory axis controlling inflammatory smooth muscle cell differentiation from stem cells and neointimal hyperplasia
Source: Stem Cell Res Ther. 2020 Nov 3;11:465. doi: 10.1186/s13287-020-01989-w (PMC7640405; doi:10.1186/s13287-020-01989-w)
Supplement: Supplementary file 2 — Additional file 2: Figure S1. Characterization of adventitia stem/progenitor cells (AdSPCs). (A) Phase-contrast image showing the primary culture of AdSPCs (P0). (B)AdSPCs at passage 3~8 were subjected to immunofluorescence staining with antibodies against AdSPC and other cell lineage markers, namely Sox10/FSP1 (fibroblast marker), Sox17/CD31 (endothelial cell marker), and Nestin/SM-NHC (SMC marker), respectively. (C) Flow cytometry analysis of AdSPC markers (Sox10 and Nestin) expression in AdSPCs at passage 3. (D) Gene expression profiles in AdSPCs at the indicated passage number (P). The data presented here are representative (A-C) or mean±S.E.M. (D) of five independent experiments. *P<0.05 (versus P3, one-way ANOVA with a post hoc test of Tukey’s analysis). [file 13287_2020_1989_MOESM2_ESM.pdf]

Figure S1. Characterization of adventitia stem/progenitor cells (AdSPCs).

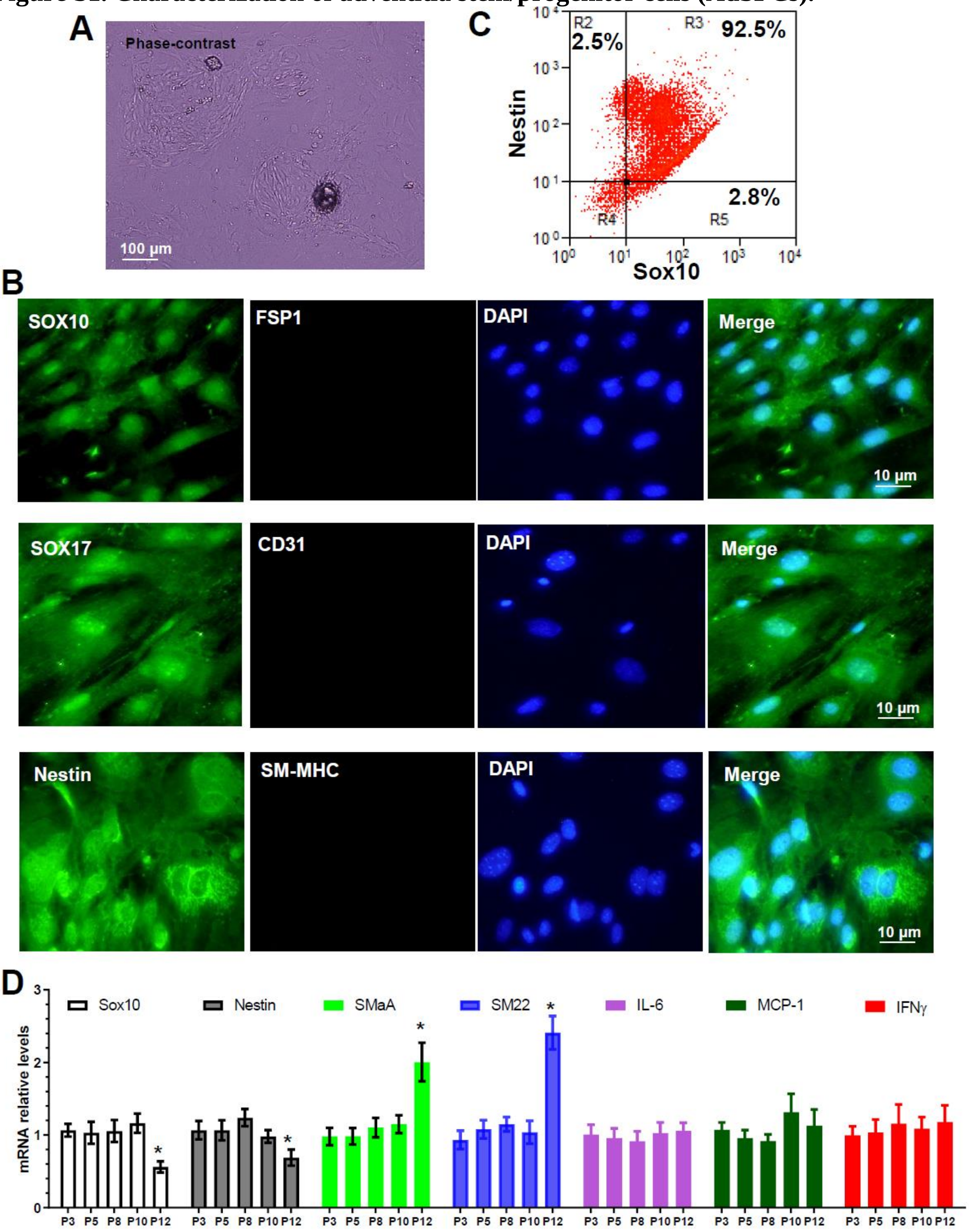

(A) Phase-contrast image showing the primary culture of AdSPCs (P0). (B)AdSPCs at passage 3~8 were subjected to immunofluorescence staining with antibodies against AdSPC and other cell lineage markers, namely Sox10/FSP1 (fibroblast marker), Sox17/CD31 (endothelial cell marker), and Nestin/SM-NHC (SMC marker), respectively. (C) Flow cytometry analysis of AdSPC markers (Sox10 and Nestin) expression in AdSPCs at passage 3. (D) Gene expression profiles in AdSPCs at the indicated passage number (P). The data presented here are representative (A-C) or mean±S.E.M. (D) of five independent experiments. *P<0.05 (versus P3, one-way ANOVA with a post hoc test of Tukey's analysis).
