# Supplementary material for: miR-214-3p-Sufu-GLI1 is a novel regulatory axis controlling inflammatory smooth muscle cell differentiation from stem cells and neointimal hyperplasia
Source: Stem Cell Res Ther. 2020 Nov 3;11:465. doi: 10.1186/s13287-020-01989-w (PMC7640405; doi:10.1186/s13287-020-01989-w)
Supplement: Supplementary file 3 — Additional file 3: Figure S2. SMC differentiation from AdSPCs. Passage 3-8 AdSPCs were cultured in SMC differentiation medium (DMEM supplemented with 5% FBS and 5ng/ml TGFβ1) for the indicated time to allow for SMC differentiation. Total RNAs and proteins from undifferentiated AdSPCs (0d) or differentiating AdSPCs at day 2, 4, 6 and 8 were harvested and subjected to RT-qPCR (A) and Western blot (B) analyses, respectively. (C) Mature SMC marker expression in AdSPC-derived SMCs. Day 8 differentiated SMCs were stained with antibodies against SMαA and SM-MHC. The data presented here are representative images (left panel in B, and C) or mean±S.E.M. (A, and right panel in B) of five independent experiments. *P<0.05 (versus 0d, one-way ANOVA with a post hoc test of Tukey’s analysis). [file 13287_2020_1989_MOESM3_ESM.pdf]

Figure S2. SMC differentiation from AdSPCs.

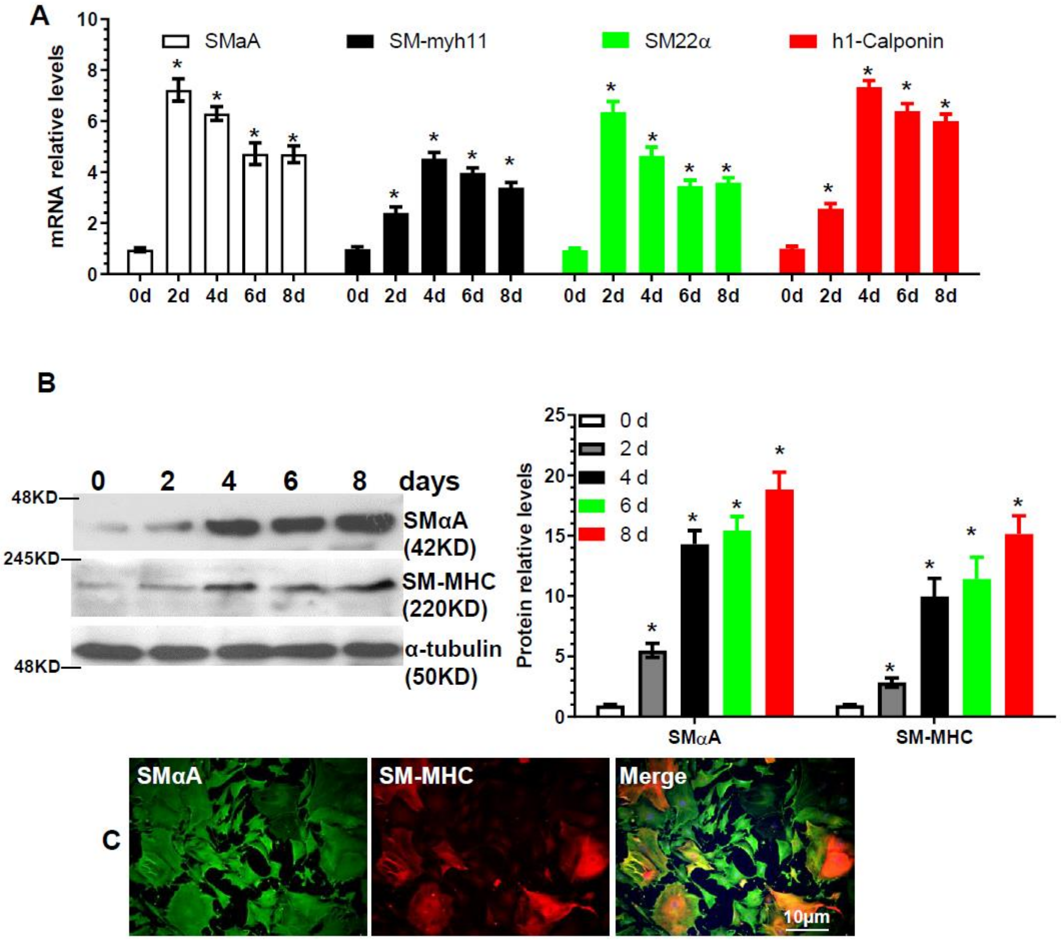

Passage 3-8 AdSPCs were cultured in SMC differentiation medium (DMEM supplemented with 5% FBS and 5ng/ml TGFβ1) for the indicated time to allow for SMC differentiation. Total RNAs and proteins from undifferentiated AdSPCs (0d) or differentiating AdSPCs at day 2, 4, 6 and 8 were harvested and subjected to RT-qPCR (A) and Western blot (B) analyses, respectively. (C) Mature SMC marker expression in AdSPC-derived SMCs. Day 8 differentiated SMCs were stained with antibodies against SMαA and SM-MHC. The data presented here are representative images (left panel in B, and C) or mean±S.E.M. (A, and right panel in B) of five independent experiments. *P<0.05 (versus 0d, one-way ANOVA with a post hoc test of Tukey's analysis).
