# Supplementary material for: miR-214-3p-Sufu-GLI1 is a novel regulatory axis controlling inflammatory smooth muscle cell differentiation from stem cells and neointimal hyperplasia
Source: Stem Cell Res Ther. 2020 Nov 3;11:465. doi: 10.1186/s13287-020-01989-w (PMC7640405; doi:10.1186/s13287-020-01989-w)
Supplement: Supplementary file 4 — Additional file 4: Figure S3. miR-214 promotes SMC differentiation from AdSPCs. (A) Increased expression of miR-214 in AdSPCs in response to TGFβ1. (B-E) miR-214 modulates SMC marker expressions. Day 2 differentiating AdSPCs were transfected with miR-214 mimics, inhibitor or respective negative control (miR ctrl), and cultured for 2~3 days, followed by RT-qPCR (B & C) and western blot (D & E) analyses, respectively. The data presented here are representative (up panels in D & E) or mean±S.E.M. (A-C, and bottom panels in D & E) of five independent experiments. *P<0.05. One-way ANOVA with a post hoc test of Tukey’s analysis for A & C, unpaired t-test for B. miR-214 indicates miR-214-3p. [file 13287_2020_1989_MOESM4_ESM.pdf]

Figure S3. miR-214 promotes SMC differentiation from AdSPCs.

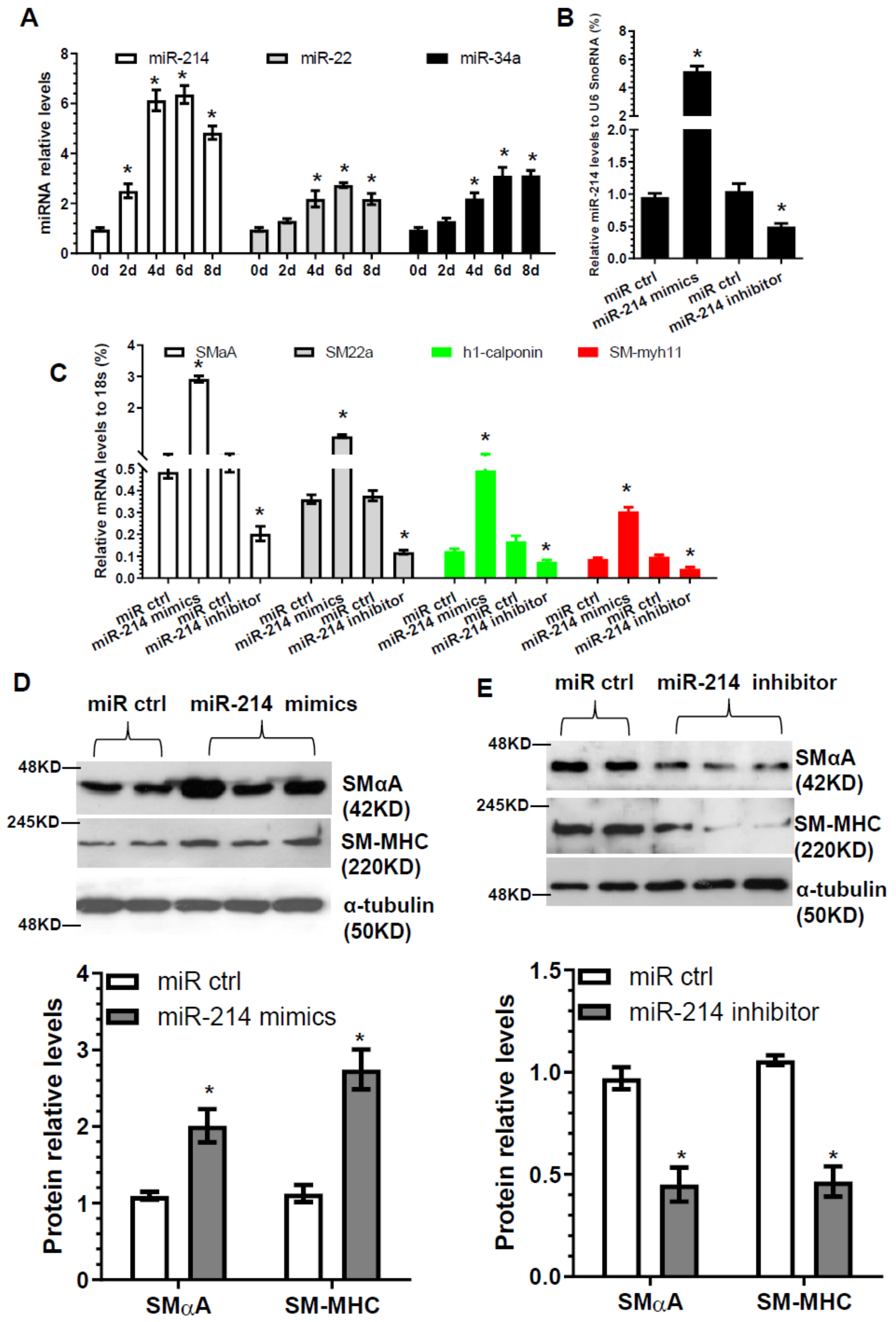

(A) Increased expression of miR-214 in AdSPCs in response to TGFβ1. (B-E) miR-214 modulates SMC marker expressions. Day 2 differentiating AdSPCs were transfected with miR-214 mimics, inhibitor or respective negative control (miR ctrl), and cultured for 2~3 days, followed by RT-qPCR (B & C) and western blot (D & E) analyses, respectively. The data presented here are representative (up panels in D & E) or mean±S.E.M. (A-C, and bottom panels in D & E) of five independent experiments. *P<0.05. One-way ANOVA with a post hoc test of Tukey's analysis for A & C, unpaired t-test for B. miR-214 indicates miR-214-3p.
