# Supplementary material for: miR-214-3p-Sufu-GLI1 is a novel regulatory axis controlling inflammatory smooth muscle cell differentiation from stem cells and neointimal hyperplasia
Source: Stem Cell Res Ther. 2020 Nov 3;11:465. doi: 10.1186/s13287-020-01989-w (PMC7640405; doi:10.1186/s13287-020-01989-w)
Supplement: Supplementary file 5 — Additional file 5: Figure S4. Characterisation of iSMC phenotype. Mature SMCs were subjected to serum starvation for 48 hours, followed by the indicated treatments for additional 24 hours. Total RNAs and conditioned culture medium harvested from SMCs treated with vehicle (serum-starved SMCs, ssSMCs), 5ng/ml TGFβ1 (‘contractile’ SMCs, conSMCs), 10ng/ml PDGF-BB (‘synthetic’ SMCs, synSMCs), and iSMCs differentiated from AdSPCs (iSMCs) were subjected to RT-qPCR (A) and ELISA analyses (B), respectively. (C) Luciferase activity assays detected NFκB activation in cells with the indicated treatments. (D) BrdU incorporation assays to detect cell proliferation. (E) Trans-well migration assays detected cell migration under 30ng/ml PDGF-BB stimulation. The data presented here are representative images (left panels in E) or mean±S.E.M. (A-D, right panel in E) of five independent experiments (n=5). *P<0.05 (versus ssSMCs), #<0.05 (versus synSMCs) (one-way ANOVA with a post hoc test of Tukey’s analysis). [file 13287_2020_1989_MOESM5_ESM.pdf]

Figure S4. Characterisation of iSMC phenotype.

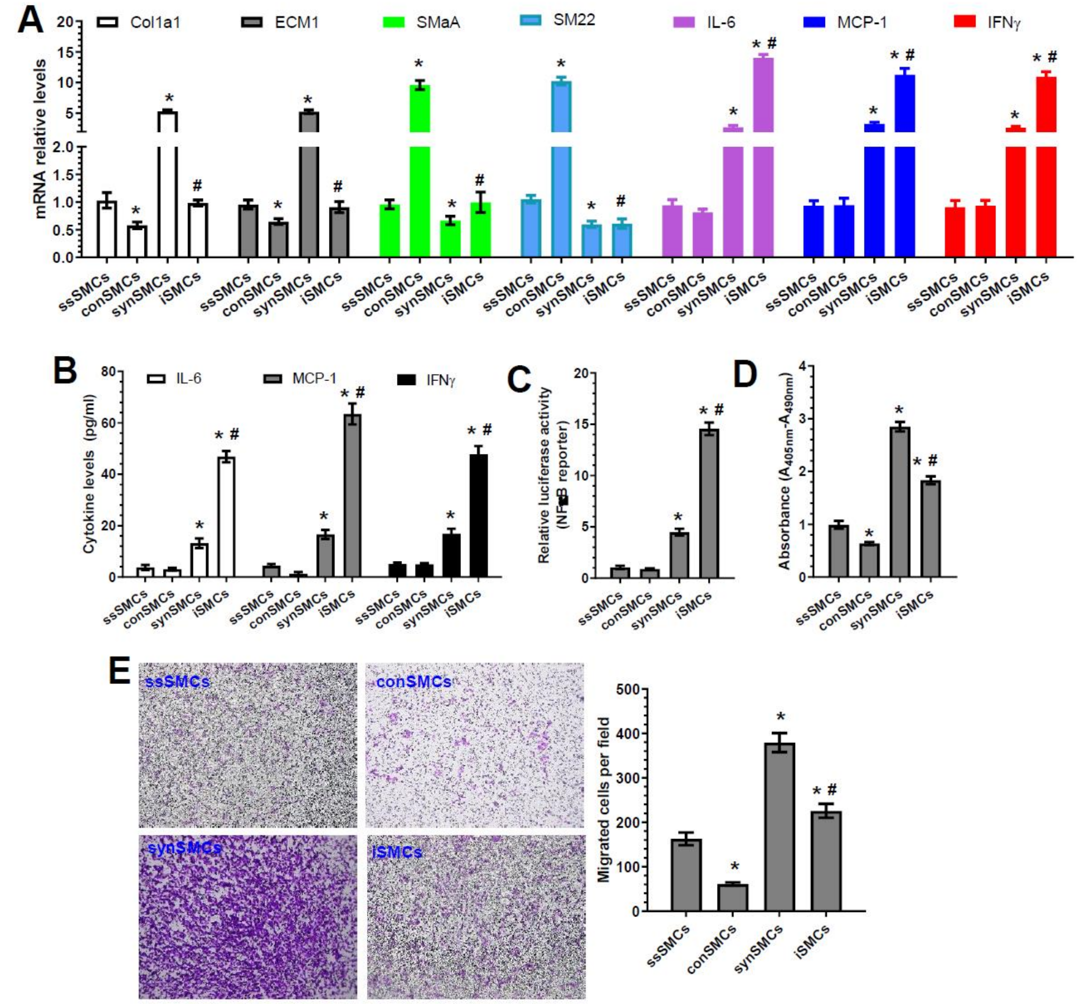

Mature SMCs were subjected to serum starvation for 48 hours, followed by the indicated treatments for additional 24 hours. Total RNAs and conditioned culture medium harvested from SMCs treated with vehicle (serum-starved SMCs, ssSMCs), 5ng/ml TGFβ1 ('contractile' SMCs, conSMCs), 10ng/ml PDGF-BB ('synthetic' SMCs, synSMCs), and iSMCs differentiated from AdSPCs (iSMCs) were subjected to RT-qPCR (A) and ELISA analyses (B), respectively. (C) Luciferase activity assays detected NFκB activation in cells with the indicated treatments. (D) BrdU incorporation assays to detect cell proliferation. (E) Trans-well migration assays detected cell migration under 30ng/ml PDGF-BB stimulation. The data presented here are representative images (left panels in E) or mean±S.E.M. (A-D, right panel in E) of five independent experiments (n=5). *P<0.05 (versus ssSMCs), $^{\#}$<0.05 (versus synSMCs) (one-way ANOVA with a post hoc test of Tukey's analysis).
