# Supplementary material for: miR-214-3p-Sufu-GLI1 is a novel regulatory axis controlling inflammatory smooth muscle cell differentiation from stem cells and neointimal hyperplasia
Source: Stem Cell Res Ther. 2020 Nov 3;11:465. doi: 10.1186/s13287-020-01989-w (PMC7640405; doi:10.1186/s13287-020-01989-w)
Supplement: Supplementary file 6 — Additional file 6: Figure S5. GLI1 cellular location was modulated by miR-214-3p. AdSPCs were differentiated into iSMCs (DMEM containing 5ng/ml TGFβ1 and 50ng/ml TNFα) for 4 days, then transfected with miR-214-3p mimics (miR-214) or negative control (miR ctrl), and cultured for 48 hours in the same culture medium. Cells were fixed and subjected to immunofluorescence staining (A). Fifty cells were randomly selected for quantification by Image J free software (Version 1.47, RRID:SCR_003070) from each treatment. Mean fluorescence intensity (MFI) of nuclear (B) and cytoplasmic (C) GLI1 (green), as well as DAPI (blue) from the same cells were quantified, and the ratio of MFI of GLI1 over DAPI were calculated accordingly. Veh indicates vehicle control for TNFα. The data presented here are representative (A) or mean±S.E.M. (B & C) of fifty cells (n=50). *P<0.05 (vs veh/miR ctrl); #P<0.05 (TNFα/miR-214 vs TNFα/miR ctrl) (one-way ANOVA with a post hoc test of Tukey’s analysis). miR-214 indicates miR-214-3p. [file 13287_2020_1989_MOESM6_ESM.pdf]

Figure S5. GLI1 cellular location was modulated by miR-214-3p.

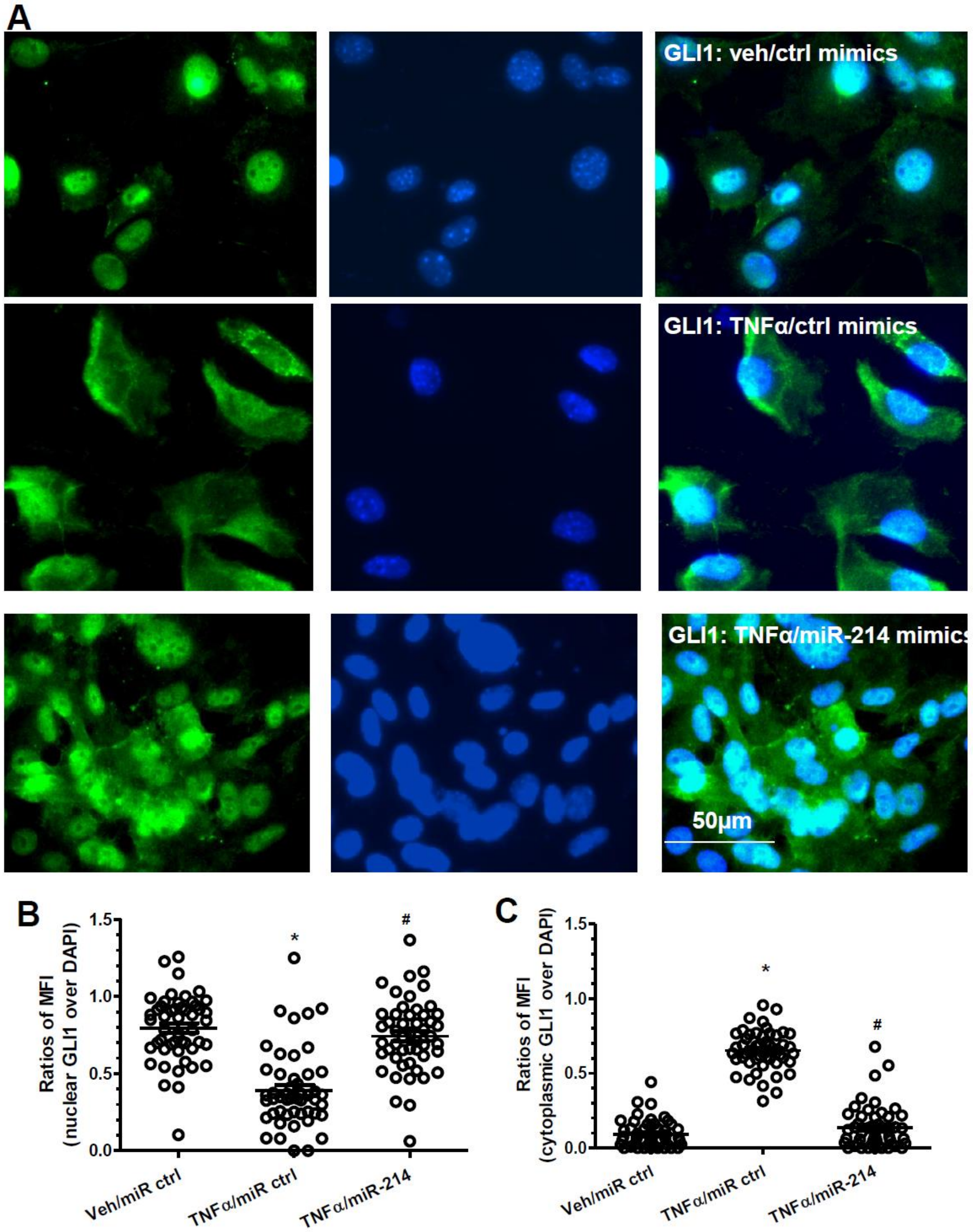

AdSPCs were differentiated into iSMCs (DMEM containing 5ng/ml TGFβ1 and 50ng/ml TNFα) for 4 days, then transfected with miR-214-3p mimics (miR-214) or negative control (miR ctrl), and cultured for 48 hours in the same culture medium. Cells were fixed and subjected to immunofluorescence staining (A). Fifty cells were randomly selected for quantification by Image J free software (Version 1.47, RRID:SCR_003070) from each treatment. Mean fluorescence intensity (MFI) of nuclear (B) and cytoplasmic (C) GLI1 (green), as well as DAPI (blue) from the same cells were quantified, and the ratio of MFI of GLI1 over DAPI were calculated accordingly. Veh indicates vehicle control for TNFα. The data presented here are representative (A) or mean±S.E.M. (B & C) of fifty cells (n=50). *P<0.05 (vs veh/miR ctrl); #P<0.05 (TNFα/miR-214 vs TNFα/miR ctrl) (one-way ANOVA with a post hoc test of Tukey's analysis). miR-214 indicates miR-214-3p.
