# Supplementary material for: miR-214-3p-Sufu-GLI1 is a novel regulatory axis controlling inflammatory smooth muscle cell differentiation from stem cells and neointimal hyperplasia
Source: Stem Cell Res Ther. 2020 Nov 3;11:465. doi: 10.1186/s13287-020-01989-w (PMC7640405; doi:10.1186/s13287-020-01989-w)
Supplement: Supplementary file 7 — Additional file 7: Figure S6. Sufu controls SMC gene expression and GLI1 cellular location. (A) SMC gene expression is negatively regulated by Sufu. AdSPCs (p3~p8) were infected with control (pCDH) or Sufu over-expression (pCDH-Sufu) lentivirus and cultured in iSMC differentiation induction medium for 2~3 days. The expression levels of a panel of SMC specific genes was examined by RT-qPCR analysis. (B) Sufu over-expression inhibited SMC gene promoter activity. AdSPCs infected with pCDH or pCDH-Sufu were transfected with SMC gene promoter reporters (pGL3-SMαA or pGL3-SM22α) as indicated. Luciferase activity assays were conducted at 48 hours post-transfection. (C) GLI1 gene expression was not affected by Sufu over-expression. (D) Sufu knockdown promotes GLI1 protein accumulation in nuclei. AdSPCs were infected with non-target (sh-NT) or Sufu (sh-Sufu) shRNA lentivirus, followed by iSMC differentiation. Cells were fixed and subjected to immunofluorescence staining with GLI1 antibody. Data presented here are representative images (D) or Mean±S.E.M of five or six independent experiments (n=5 or 6, unpaired t-test). [file 13287_2020_1989_MOESM7_ESM.pdf]

Figure S6. Sufu controls SMC gene expression and GLI1 cellular location.

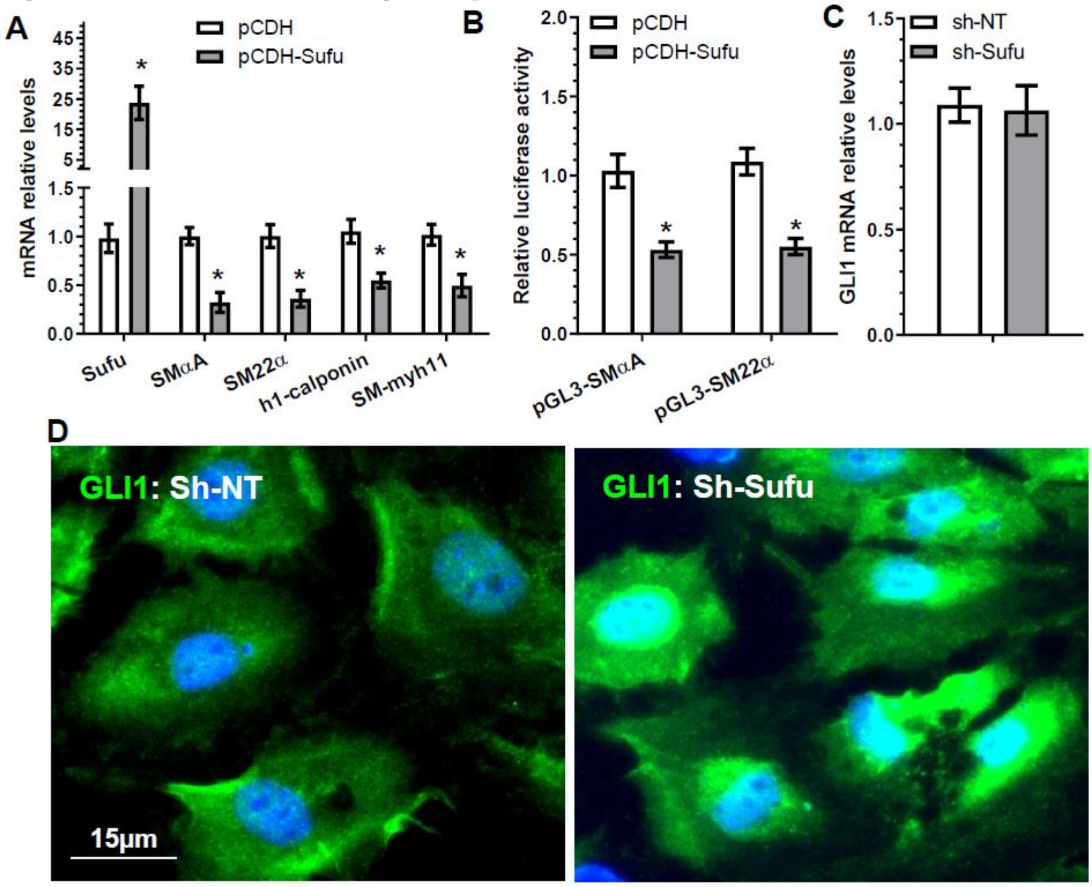

(A) SMC gene expression is negatively regulated by Sufu. AdSPCs (p3~p8) were infected with control (pCDH) or Sufu over-expression (pCDH-Sufu) lentivirus and cultured in iSMC differentiation induction medium for 2~3 days. The expression levels of a panel of SMC specific genes was examined by RT-qPCR analysis. (B) Sufu over-expression inhibited SMC gene promoter activity. AdSPCs infected with pCDH or pCDH-Sufu were transfected with SMC gene promoter reporters (pGL3-SMαA or SM22α) as indicated. Luciferase activity assays were conducted at 48 hours post-transfection. (C) GLI1 gene expression was not affected by Sufu over-expression. (D) Sufu knockdown promotes GLI1 protein accumulation in nuclei. AdSPCs were infected with non-target (sh-NT) or Sufu (sh-Sufu) shRNA lentivirus, followed by iSMC differentiation. Cells were fixed and subjected to immunofluorescence staining with GLI1 antibody. Data presented here are representative images (D) or Mean±S.E.M of five or six independent experiments (n=5 or 6, unpaired t-test).
