# Supplementary material for: miR-214-3p-Sufu-GLI1 is a novel regulatory axis controlling inflammatory smooth muscle cell differentiation from stem cells and neointimal hyperplasia
Source: Stem Cell Res Ther. 2020 Nov 3;11:465. doi: 10.1186/s13287-020-01989-w (PMC7640405; doi:10.1186/s13287-020-01989-w)
Supplement: Supplementary file 8 — Additional file 8: Figure S7. Sufu controls SMC gene expression through regulating SRF. (A-B) Serum response factor (SRF) binding sites within SMC gene promoters are required for Sufu-mediated SMC gene repression. AdSPCs infected with non-target (sh-NT) or Sufu (sh-Sufu) shRNA lentivirus were transfected with wild type (WT) SMC gene promoter reporters (pGL3-SMαA or pGL3-SM22α), or SRF binding site mutants [SRFmut], respectively. Luciferase activity assay were measured at 48 hours post-transfection. (C-D) Sufu over-expression inhibited SRF gene expression, while Sufu knockdown promoted its expression. (E) SRF gene promoter activity is increased by Sufu inhibition. (F) GLI1 binding site within SRF gene promoter region is necessary for SRF gene up-regulation by Sufu knockdown in AdSPCs. The potential wild type binding site (pGL3-SRF-WT) of GLI1 within SRF gene promoter and its mutant pGL3-SRF-GLI1mut) are depicted in this illustration (Insert). AdSPCs infected with sh-NT or sh-Sufu lentivirus were transfected with respective SRF gene promoter reporters as indicated. Luciferase activity assay were measured at 48 hours post-transfection. (G) Sufu inhibition increases GLI1 enrichment within SRF gene promoter. ChIP assays were performed using antibody against GLI1 or normal IgG, respectively. PCR amplifications of the adjacent regions were included as additional control for specific promoter DNA enrichment. Data presented here are Mean±S.E.M of five or six independent experiments (n=5 or 6, unpaired t-test). [file 13287_2020_1989_MOESM8_ESM.pdf]

Figure S7. Sufu controls SMC gene expression through regulating SRF.

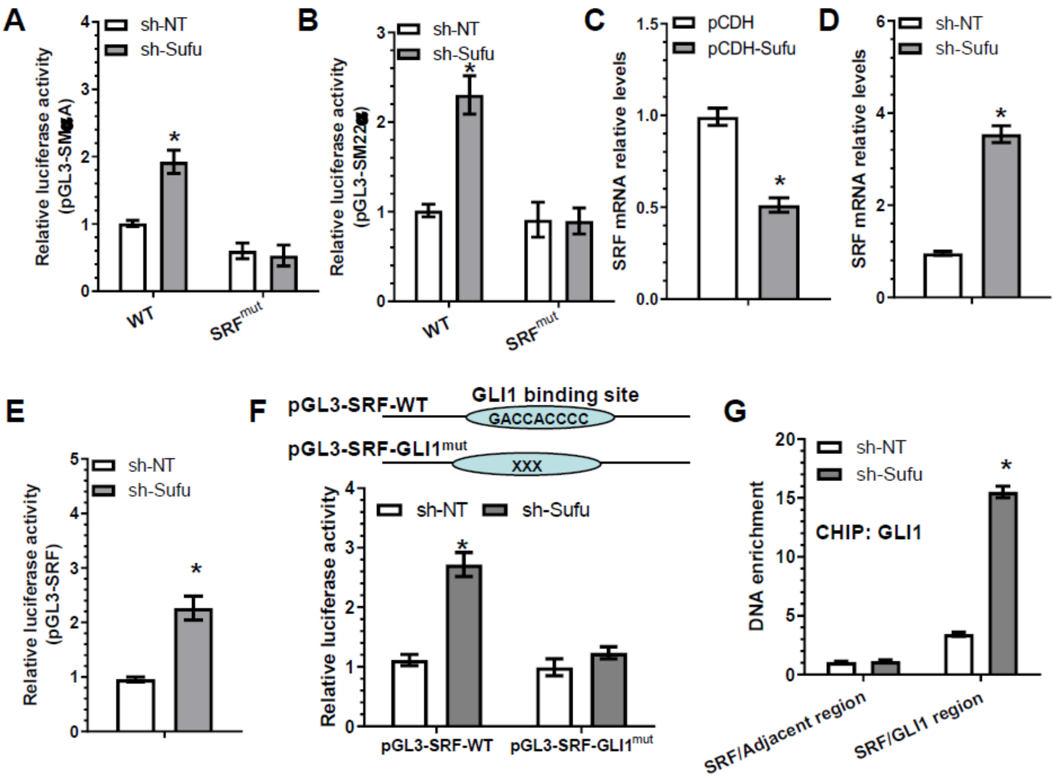

(A-B) Serum response factor (SRF) binding sites within SMC gene promoters are required for Sufu-mediated SMC gene repression. AdSPCs infected with non-target (sh-NT) or Sufu (sh-Sufu) shRNA lentivirus were transfected with wild type (WT) SMC gene promoter reporters (pGL3-SMαA or SM22α), or SRF binding site mutants [SRF^{mut}], respectively. Luciferase activity assay were measured at 48 hours post-transfection. (C-D) Sufu over-expression inhibited SRF gene expression, while Sufu knockdown promoted its expression. (E) SRF gene promoter activity is increased by Sufu inhibition. (F) GLI1 binding site within SRF gene promoter region is necessary for SRF gene up-regulation by Sufu knockdown in AdSPCs. The potential wild type binding site (pGL3-SRF-WT) of GLI1 within SRF gene promoter and its mutant pGL3-SRF-$GLI1^{mut}$) are depicted in this illustration (Insert). AdSPCs infected with sh-NT or sh-Sufu lentivirus were transfected with respective SRF gene promoter reporters as indicated. Luciferase activity assay were measured at 48 hours post-transfection. (G) Sufu inhibition increases GLI1 enrichment within SRF gene promoter. ChIP assays were performed using antibody against GLI1 or normal IgG, respectively. PCR amplifications of the adjacent regions were included as additional control for specific promoter DNA enrichment. Data presented here are Mean±S.E.M of five or six independent experiments (n=5 or 6, unpaired t-test).
