# Supplementary material for: miR-214-3p-Sufu-GLI1 is a novel regulatory axis controlling inflammatory smooth muscle cell differentiation from stem cells and neointimal hyperplasia
Source: Stem Cell Res Ther. 2020 Nov 3;11:465. doi: 10.1186/s13287-020-01989-w (PMC7640405; doi:10.1186/s13287-020-01989-w)
Supplement: Supplementary file 9 — Additional file 9: Figure S8. miR-214 decreases iSMCs during arterial remodelling. After wire-induced femoral arterial injury, 100 μl of 30% pluronic gel containing 2.5 nmol control AgomiR (Cel-miR-67) or miR-214 AgomiR (miR-214) per artery per mouse was immediately applied and packed around the injured femoral arteries. Two weeks later, injured segments of femoral arteries were harvested for H&E staining analyses. Mice underwent a sham arterial injury procedure serve as control (Sham). SMCs and neointima (NI) cells were isolated from media of sham mice (SMCs/Sham) or media/neointima layers of injured mice received control (NI cells/Cel-miR-67) or miR-214 (NI cells/miR-214) AgomiR, respectively. Isolated cells were cultured in SMC medium for 5-7 days and subjected to various analyses as indicated. (B) Phase-contrast images were also taken prior to other analyses and included here. (C & D) Immunofluorescence staining assays with antibodies against SMαA and IL-6. Representative images (C) and percentage of iSMCs from each group ((D) were presented here. (E) RT-qPCR detection of gene expression. Data presented here are representative images or Mean±S.E.M of five independent experiments (n=5). *P<0.05 (versus sham), #P<0.05 (miR-214 versus Cel-miR-67) (one-way ANOVA with a post hoc test of Tukey’s analysis). miR-214 indicates miR-214-3p. [file 13287_2020_1989_MOESM9_ESM.pdf]

Figure S8. miR-214 decreases iSMCs during arterial remodelling.

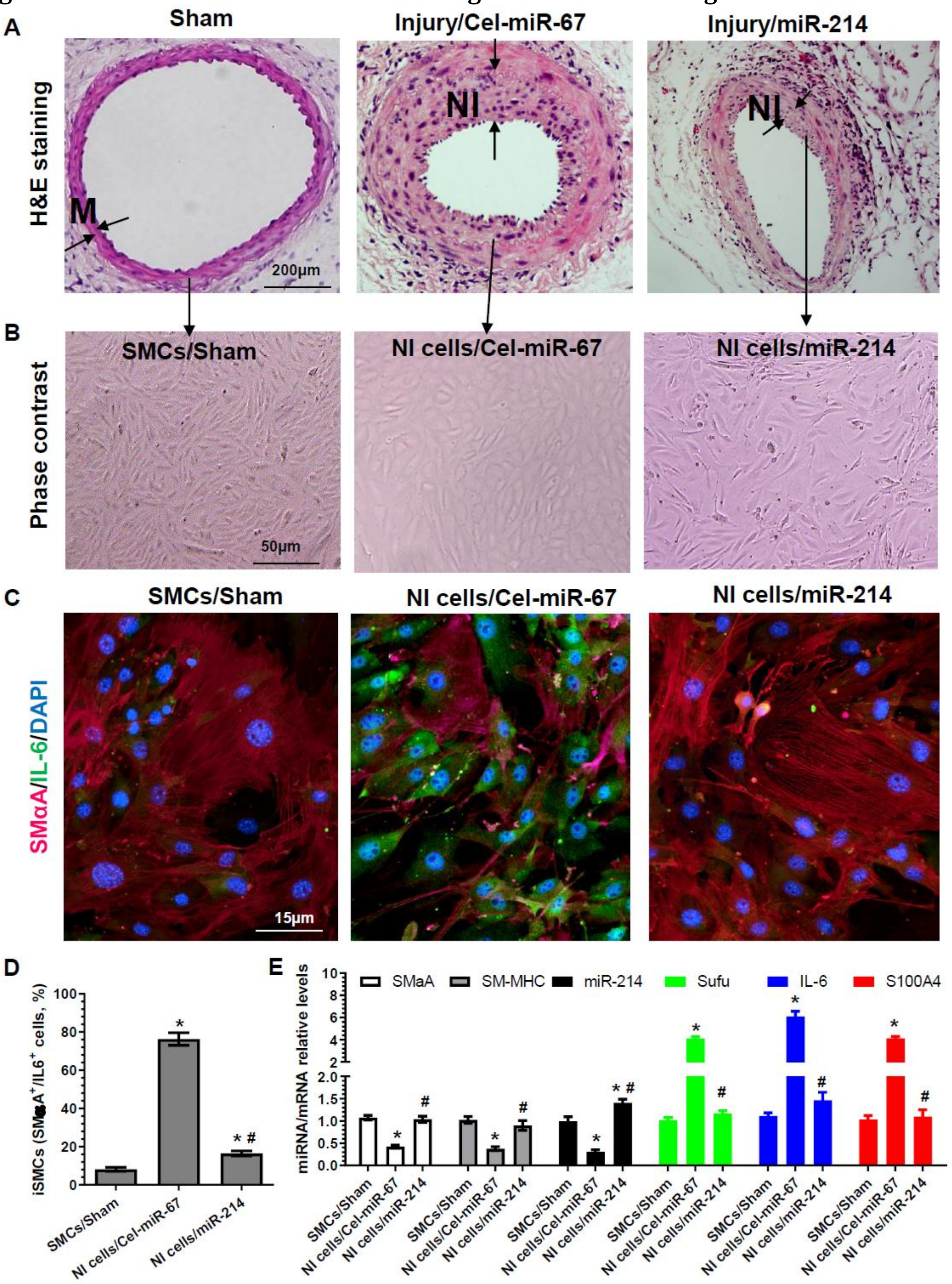

After wire-induced femoral arterial injury, 100 µl of 30% pluronic gel containing 2.5 nmol control AgomiR (Cel-miR-67) or miR-214 AgomiR (miR-214) per artery per mouse was immediately applied and packed around the injured femoral arteries. Two weeks later, injured segments of femoral arteries were harvested for H&E staining analyses. Mice underwent a sham arterial injury procedure serve as control (Sham). SMCs and neointima (NI) cells were isolated from media of sham mice, (SMCs/Sham) or media/neointima layers of injured mice received control (NI cells/Cel-miR-67) or miR-214 (NI cells/miR-214) AgomiR, respectively. Isolated cells were cultured in SMC medium for 5-7 days and subjected to various analyses as indicated. (B) Phase-contrast images were also taken prior to other analyses and included here. (C & D) Immunofluorescence staining assays with antibodies against SMαA and IL-6.

Representative images (C) and percentage of iSMCs from each group ((D) were presented here. (E) RT-qPCR detection of gene expression. Data presented here are representative images or Mean±S.E.M of five independent experiments (n=5). *P<0.05 (versus sham), #P<0.05 (miR-214 versus Cel-miR-67) (one-way ANOVA with a post hoc test of Tukey's analysis). miR-214 indicates miR-214-3p.
