# Supplementary material for: miR-214-3p-Sufu-GLI1 is a novel regulatory axis controlling inflammatory smooth muscle cell differentiation from stem cells and neointimal hyperplasia
Source: Stem Cell Res Ther. 2020 Nov 3;11:465. doi: 10.1186/s13287-020-01989-w (PMC7640405; doi:10.1186/s13287-020-01989-w)
Supplement: Supplementary file 10 — Additional file 10: Figure S9. Transplanted AdSPCs differentiate toward iSMCs during arterial remodelling. After injury, 100μl of Geltrex contained 1x10^6^ AdSPCs infected with Lenti-GFP per vessel per mice was immediately applied and packed around injured vessel. One week later, injured segments of femoral arteries were harvested and prepared, followed by triple immunofluorescence staining with antibodies against GFP, SMαA and S100A4. White arrows within merged image indicate iSMCs derived from transplanted AdSPCs. Data presented here are representative images from three mice. [file 13287_2020_1989_MOESM10_ESM.pdf]

Figure S9. Transplanted AdSPCs differentiate toward iSMCs during arterial remodelling.

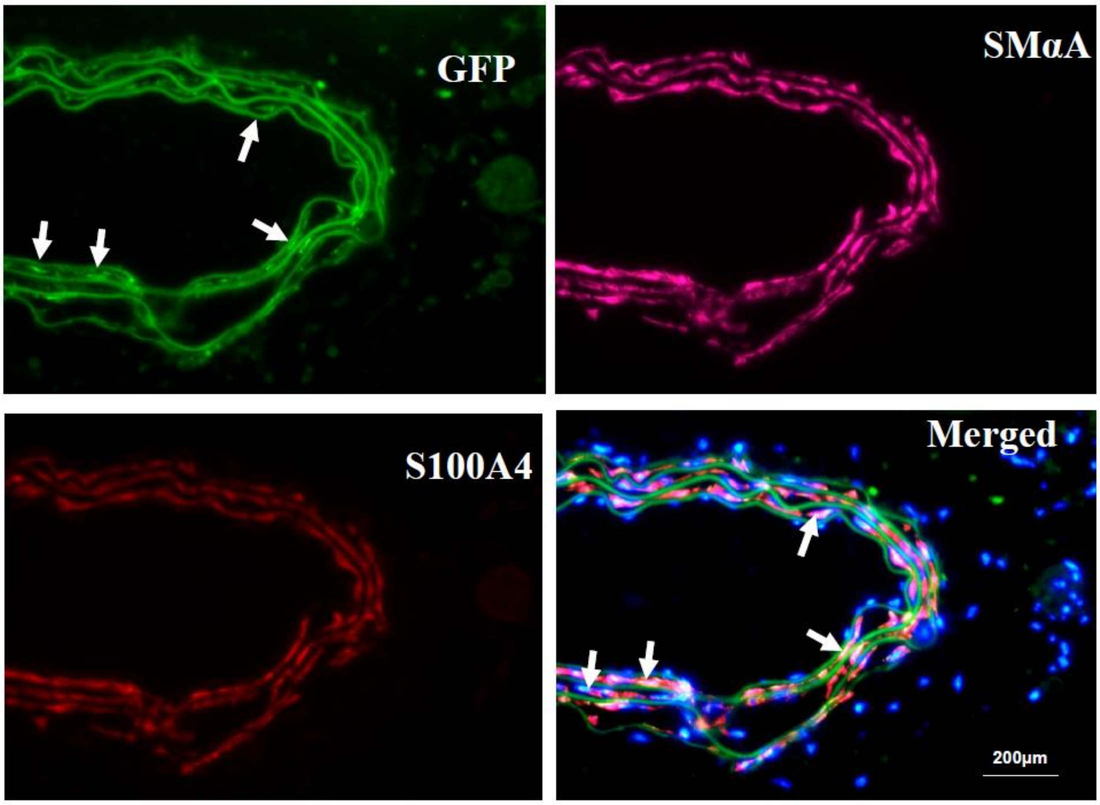

After injury, 100μl of Geltrex contained $1x10^6$ AdSPCs infected with Lenti-GFP per vessel per mice was immediately applied and packed around injured vessel. One week later, injured segments of femoral arteries were harvested and prepared, followed by triple immunofluorescence staining with antibodies against GFP, SMαA and S100A4. White arrows within merged image indicate iSMCs derived from transplanted AdSPCs. Data presented here are representative images from three mice.
